# GPR35-mediated metabolic reprogramming promotes tumorigenesis in digestive cancers

**DOI:** 10.3389/fimmu.2025.1668471

**Published:** 2025-12-12

**Authors:** Fang Wang, Xin-Xin Ding, Tao-Hong Su, Jing Cao, Feng-Qin Li, Zi-Shu Dong, Xin-Zhi Guo, Yan Zhang, Yang Chen, Dong-Hua Yang, Ping Luo

**Affiliations:** 1 Cancer Research Center, The Jiangxi Province Key Laboratory for Diagnosis, Treatment, and Rehabilitation of Cancer in Chinese Medicine, Jiangxi University of Chinese Medicine, Nanchang, China; 2 School of Pharmacy, Jiangxi University of Chinese Medicine, Nanchang, China; 3 State Key Laboratory of Phytochemistry and Natural Medicines, Dalian Institute of Chemical Physics, Chinese Academy of Sciences, Dalian, China; 4 New York College of Traditional Chinese Medicine, Mineola, NY, United States

**Keywords:** GPR35, metabolic reprogramming, digestive cancers, tumor microenvironment, gut microbiota

## Abstract

G protein-coupled receptor 35 (GPR35), a member of the largest druggable gene family, has emerged as a critical regulator of tumor metabolism and immune modulation. Aberrant expression of GPR35 is frequently observed in digestive system malignancies and is associated with poor prognosis. This review comprehensively explores GPR35’s role in metabolic reprogramming, highlighting its regulatory functions in glucose, lipid, amino acid, and microbial metabolite metabolism. GPR35 shapes the tumor microenvironment through modulation of metabolite signaling, influencing angiogenesis, immune cell infiltration, and inflammation. It also acts as a key interface between host cells and the gut microbiota, contributing to cancer progression via microbial-derived metabolites. Pharmacological targeting of GPR35 shows promise, with several agonists and antagonists advancing through preclinical and early clinical development. However, challenges such as species-specific pharmacodynamics, ligand selectivity, and receptor isoform variability complicate drug development. Recent advances, including the creation of humanized GPR35 models, have facilitated translational research. Targeting GPR35-mediated metabolic reprogramming represents a novel therapeutic strategy, particularly for metabolically active digestive cancers. Future studies should focus on clarifying the metabolic pathways governed by GPR35 and optimizing receptor-specific therapeutics for clinical application.

## 1 Introduction

Digestive cancers represent a major global health burden, with significant increases in incidence and mortality projected across nearly all regions worldwide (1). These malignancies originate within the gastrointestinal tract and encompass a broad spectrum of diseases, including colorectal cancer (CRC), hepatocellular carcinoma (HCC), gastric cancer (GC), and pancreatic ductal adenocarcinoma (PDAC), among others (2, 3). Early detection remains challenging due to the limited sensitivity of current screening methods and a high degree of biological heterogeneity, that collectively contribute to poor prognoses and elevated mortality rates (4). This variability necessitates the development of tailored strategies for both diagnosis and treatments. According to the World Health Organization, digestive cancers account for over one-quarter of the global cancer burden, comprising 26% of all newly diagnosed cancer cases. With global populations expanding and aging, the incidence of digestive cancers is expected to rise significantly, reaching an estimated 7.5 million new cases annually by 2040 (5). In several high-income countries, cancer has surpassed cardiovascular disease as the leading cause of death, with digestive cancers contributing disproportionately to this trend (6). Consequently, the development of effective strategies for prevention, early detection and treatment is an urgent global health priority.

The study of cancer metabolism predates the discovery of oncogenes and tumor suppressors by several decades, and recent research continues to underscore its fundamental role in cancer progression, particularly in tumors of digestive system (7–10). Cancer cells undergo profound metabolic reprogramming to sustain uncontrolled proliferation, invasion and resistance to therapy (11–14). These alterations involve the dysregulation of glucose, amino acid, and lipid metabolism, along with complex interactions with the gut microbiome (15). A classical hallmark of this metabolic shift is the Warburg effect, wherein cancer cells preferentially rely on aerobic glycolysis and glutamine metabolism for rapid energy production, even under oxygen-rich conditions (16). For example, the glycolytic enzyme ENO1 undergoes O-GlcNAcylation at threonine 19, promoting dimerization and enhancing glycolytic flux to support tumor growth (17). Similarly, overexpression of glucose transporters, such as sodium-glucose cotransporter 2 and glucose transporter 1 (GLUT1), contributes to metabolic reprogramming and tumorigenesis in HCC (18). Bone morphogenetic protein 4, a key regulator of adipogenesis in obesity and diabetes, promotes glycogen synthesis and HCC progression via the small mother against decapentaplegic/solute carrier family 2 member 1 signaling pathway (19). Collectively, these findings underscore the essential role of metabolic dysregulation in tumor development and progression, highlighting its value as a therapeutic target.

GPR35, a rhodopsin-like orphan G protein-coupled receptor (GPCR), has recently emerged as a key modulator of both metabolic regulation and immune signaling. GPR35 is widely expressed, particularly in the digestive system, and participates in diverse physiological functions, including metabolic homeostasis, intestinal barrier integrity, inflammation, and immune modulation (20–22). Notably, GPR35 regulates multiple metabolic pathways, including lipid, glucose, and amino acid metabolism. Studies in GPR35 knockout mice have demonstrated increased body weight, elevated hepatic triglyceride levels, and exacerbated steatohepatitis (23). Upon activation, GPR35 triggers intracellular signaling cascade, including Ca^2+^ mobilization, phosphorylation of extracellular signal-regulated kinases 1 and 2 (ERK1/2), and activation of peroxisome proliferator-activated receptor gamma coactivator 1alpha (PGC-1α), thereby accelerating lipid metabolism (21, 24). GPR35 is instrumental in regulating hepatocellular lipid synthesis and mediating signaling through pathways, such as the p38 mitogen-activated protein kinase (MAPK) and c-Jun N-terminal kinase (JNK). The GPR35 agonist lodoxamide has been shown to inhibit hepatic lipid accumulation, indicating its potential as a therapeutic agent for nonalcoholic fatty liver disease (NAFLD) (25). In glycolytic regulation, GPR35 is essential for sustaining energy metabolism and proliferation in intestinal epithelial cells by interacting with the α-subunit of the sodium-potassium pump, promoting ion transport, activating Src kinase, and increasing cellular glucose demand (26). In amino acid metabolism, GPR35 also plays a crucial role by sensing kynurenic acid (KYNA), a critical metabolite in the tryptophan metabolic pathway, particularly in contexts such as chemotherapy-induced intestinal injury (27).

While recent reviews have comprehensively summarized the molecular basis of GPR35 in cancer and immunity, as well as advances in its therapeutic targeting (28, 29), a focused examination of its role in the metabolic reprogramming of digestive system tumors remains unexplored. Once considered an “orphan receptor”, GPR35 has now emerged as a pivotal player in gastrointestinal cancer biology. The network of interactions between GPR35 and its endogenous ligands, and how they precisely regulate oncogenic signal transduction, constitutes an active and critical frontier at the cellular level (28, 29). It is this multifunctionality that provides a compelling rationale for its distinct role in physiology and pathophysiology compared to other GPCRs, solidifying its potential as a promising oncology target, emerging evidence have identified GPR35 as a critical regulatory factor in digestive cancers. For instance, Macrophage-specific GPR35 deletion attenuates colitis-associated and spontaneous colon tumor formation by disrupting Na^+^/K^+^-ATPase (NKA)/Src-mediated angiogenesis and extracellular matrix remodeling (30). In gastric cancer, enhancer release and relocalization (ERR) activates GPR35 expression in tumor tissues, promoting disease progression and negatively impacting patient prognosis (31). GPR35 also facilitates intestinal tumorigenesis by enhancing glycolysis through its interaction with NKA (26). In CRC, modulation of GPR35 expression affects fatty acid β-oxidation and phosphatidylethanolamine metabolism, indicating its pivotal role in tumor metabolic regulation (32). As research continues to unravel the dual role of GPR35 in metabolic control and oncogenesis, the interplay between GPR35-mediated metabolic adaptations and cancer progression is gaining increasing attention. This review synthesizes current findings on the role of GPR35 in cancer biology and metabolism and highlights its potential as a therapeutic target for metabolic reprogramming in digestive cancers.

## 2 Methods

This review encompassed all records from the inception of the databases to November 2025, using keywords including “GPR35”, “cancer”, “metabolic reprogramming”, “ligand”, “tumor microenvironment”, “digestive cancers” and “gut microbiota”. The selected databases included PubMed (https://pubmed.ncbi.nlm.nih.gov), Web of Science (http://apps.webofknowledge.com/), China National Knowledge Infrastructure (http://www.cnki.net), and Elsevier ScienceDirect (https://www.sciencedirect.com/).

The inclusion criteria for this review encompassed studies related to the expression, function, and molecular mechanisms of GPR35 in digestive cancers (including gastric cancer, liver cancer, pancreatic cancer, and colorectal cancer); studies specifically focusing on how GPR35 influences the occurrence and development of digestive cancers through the regulation of metabolic reprogramming (such as glucose metabolism, lipid metabolism, amino acid metabolism, etc.); and research involving ligands, agonists, or antagonists targeting GPR35 and their potential applications in cancer therapy. All published studies in English and Chinese, including basic research, *in vitro* and *in vivo* experiments, and relevant preclinical studies, were included without any language restrictions. The literature screening process initially involved a preliminary selection based on article titles, followed by a secondary screening through abstract review. Finally, the full texts of the eligible studies were thoroughly read and analyzed in detail.

## 3 The expression pattern and function of GPR35

GPR35 is a rhodopsin-like, seven-transmembrane domain receptor and a member of the class A GPCR family. In humans, GPR35 gene exists in two splice variants: GPR35a and GPR35b, comprising 309 and 340 amino acids, respectively. According to recent cryo-electron microscopy studies, GPR35 shares structural features common to GPCRs, including three extracellular loops, three intracellular loops, and a characteristic amphiphilic helical transmembrane domain (33). It contains two conserved motifs, NPXXY and CWXP, which are critical for their function. While nonconservative substitutions occur in the NPXXY motif, substitutions in the CWXP motif are more conserved across species (33, 34). Sequence similarity between human GPR35a and its mouse and rat homologs is approximately 72% and 73%, respectively (35), with species-specific differences noted in GPR35 phosphorylation and activation mechanisms (36).

GPR35 is broadly expressed across multiple organ systems, with particularly high levels in the small intestine and colon, as well as in the stomach, liver, spleen, kidneys, and sympathetic neurons (35, 37). This expression profile suggests a significant role in maintaining gastrointestinal homeostasis. At the cellular level, GPR35 is expressed in various immune cell types, including monocytes, T cells, neutrophils, dendritic cells, and invariant natural killer T cells, implicating its role in immune responses (38, 39).

Although GPR35 is classified as an orphan receptor due to the lack of a universally accepted endogenous ligand, several both endogenous and exogenous molecules, have been shown to activate it and elicit physiological responses. Early candidates include lysophosphatidic acid (LPA) (40), KYNA (41) and C-X-C motif chemokine ligand 17 (CXCL17) (42). Although the classification of CXCL17 as a GPR35 ligand remains controversial, recent studies demonstrate that the CXCL17-GPR35 axis mediates myeloid-derived suppressor cell recruitment during liver ischemia-reperfusion injury (43) and activates IL-17 signaling to promote chemoresistance in CRC (44). KYNA has been shown to activate GPR35 in a dose-dependent manner, attenuate acetic acid-induced writhing in mice, and alleviate inflammatory pain (45). More recently, 5-hydroxyindoleacetic acid (5-HIAA) has been identified as a novel ligand for GPR35, capable of activating both human and rodent receptors at nanomolar concentrations and promoting neutrophil chemotaxis (46). Additionally, specific breast milk oligosaccharides, such as 3’-O-sialic acid lactose and 6’-O-sialic acid lactose, have been reported to activate GPR35 (47). While debate continues over the definitive identity of endogenous ligands, the broad physiological effects of GPR35 underscore its therapeutic relevance, particularly in digestive system disorders.

## 4 Role of GPR35 in digestive cancers

Following the preliminary elucidation of GPR35’s receptor characteristics, tissue distribution, and complex ligand repertoire, the pathological roles and underlying mechanisms it plays in various digestive tract malignancies have emerged as a pivotal scientific issue requiring urgent investigation. This section will focus on the specific signal transduction mechanisms of GPR35 in digestive tract cancers, systematically examining how it regulates key downstream pathways to influence tumor cell proliferation, apoptosis, invasion, migration, and tumor microenvironment remodeling, thereby profoundly contributing to the pathogenesis of these cancers.

### 4.1 GPR35 in hepatocellular carcinoma

**Figure 1:**
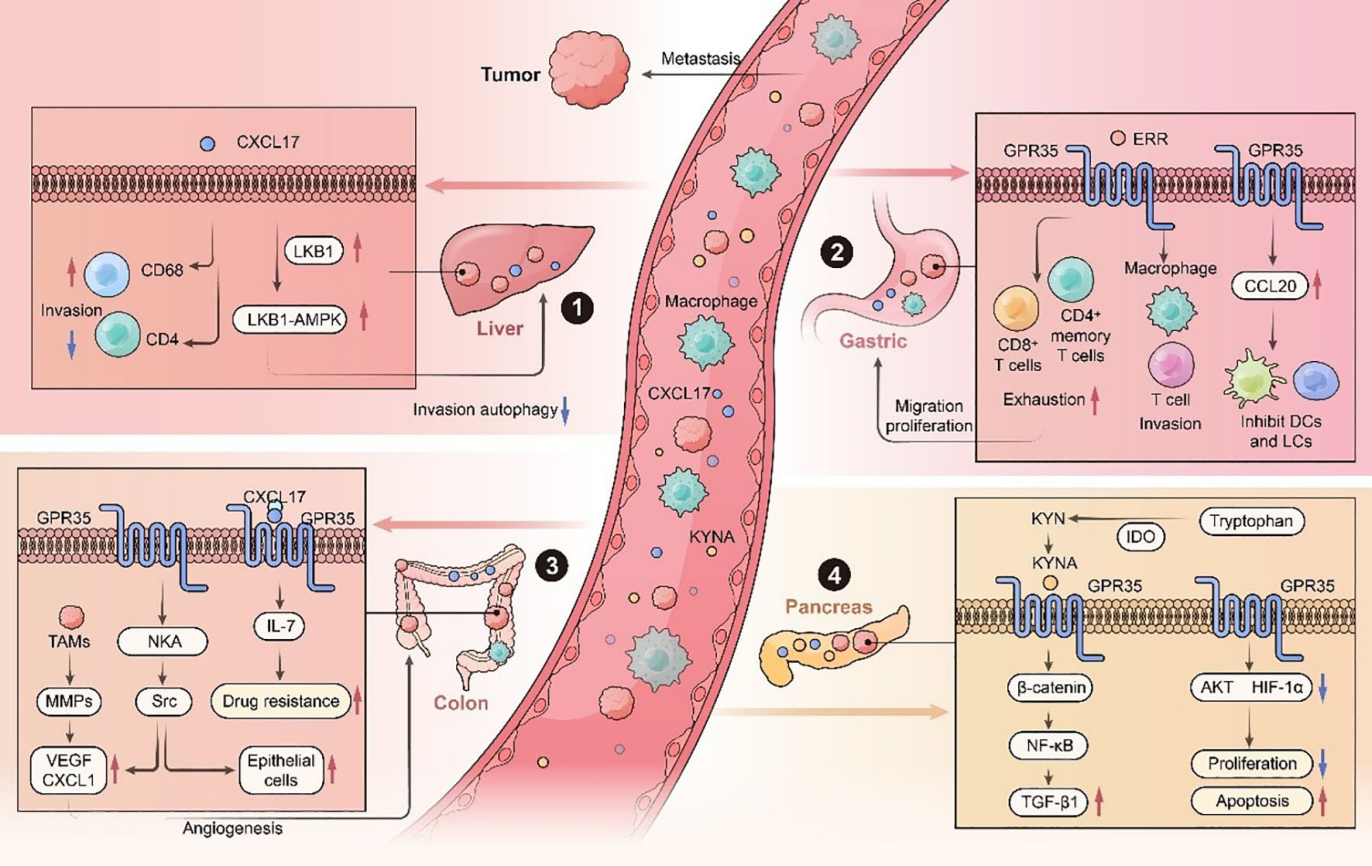
Role of GPR35 and its endogenous ligands in digestive cancers. GPR35 exerts diverse and critical roles across multiple malignancies by mediating oncogenic signaling pathways. (1) In HCC, CXCL17 enhances metastatic by activating the LKB1-AMPK pathway while concurrently suppressing autophagic flux. (2) In GC, ERR-mediated upregulation of GPR35 fosters an immunosuppressive tumor microenvironment by inducing macrophage M2 polarization and T-cell exhaustion. (3) GPR35 activation promotes angiogenesis and chemoresistance in CRC through IL-17 signaling. (4) In PDAC, GPR35 enhances tumor progression by kynurenine (KYN) pathway-mediated TGF-β1 upregulation, while paradoxically inhibiting proliferation via decreased AKT and HIF-1α (hypoxia-inducible factor-1 alpha) phosphorylation. LiverkinaseB1: LKB1, Na^+^/K^+^-ATPase: NKA, Matrix Metalloproteinases: MMPs, Tumor-associated macrophages: TAMs, Enhancer release and retargeting: ERR, Vascular endothelial growth factor: VEGF, C-X-C motif chemokine ligand 17: CXCL17.

Hepatocellular carcinoma is the third leading cause of cancer-related death worldwide and ranks fifth among men and seventh among women in global cancer mortality (1, 48), with particularly high incidence rates in East Asia (49). Despite advancements in prevention and treatment, the prognosis for HCC remains poor, with fewer than 20% of patients surviving beyond five years. GPR35, due to its pleiotropic biological functions, has been implicated in the complex pathogenesis of HCC. High CXCL17 expression in HCC tissues is associated with poor prognosis (**Table 1**). CXCL17 promotes the infiltration of CD4^+^ T cells and CD68^+^ immune cells into the tumor microenvironment, thereby accelerating tumor growth and metastasis (50). Additionally, CXCL17 directly enhances HCC cell proliferation and migration, contributing to malignant progression (51). This tumor-promoting role is further supported by CXCL17’s ability to inhibit autophagy and activate the LKB1-AMPK signaling pathway while modulating immune cell infiltration (51) (**Figure 1**).

**Table 1:** Role of GPR35 in digestive cancers.

Cancer	Role	Refs
HCC	CXCL17 triggers malignant progression of HCC	(50, 51)
GC	Promotes GC progression	(31, 52)
	Highly expressed in tumor regions of GC patients and may be involved in GC formation	(53)
CRC	Associated with poor prognosis in CRC patients	(54)
	Drives CRC tumorigenesis and chemoresistance	(44)
	Activates Src and promotes intestinal epithelial cell proliferation	(26, 30)
	Promotes CRC angiogenesis and metastasis	(55)
	GPR35 antagonist CID-2745687 inhibits CRC cells growth	(56)
Pancreatic cancer	A predicted pivotal therapeutic targets pancreatic adenocarcinoma.	(57)
	Regulates the proliferation, migration, and invasion of pancreatic cancer cells, Predicts poor prognosis	(54, 58)
	CXCL17 promotes anti-tumor immunity in the early stages of pancreatic cancer	(59)

Beyond its role in the CXCL17 axis, GPR35 is also critical for hepatic lipid metabolism. Spatial multi-omics analysis has shown that GPR35 knockout mice fed a high-fat diet exhibit excessive weight gain, exacerbated nonalcoholic fatty liver disease, and increased hepatic triglyceride accumulation (23), all established risk factors for HCC development. These findings suggest that GPR35 may promote the pathogenesis and metastasis of HCC through CXCL17-mediated oncogenic signaling. Interestingly, GPR35 also appears to play a protective role in hepatic lipid metabolism. This dual function highlights the complexity of GPR35 in liver pathophysiology, and further studies are needed to clarify the underlying mechanisms.

### 4.2 GPR35 in gastric cancer

Gastric cancer is one of the most common malignant tumors of the digestive system and ranks as the fifth most prevalent cancer globally (1, 60). Patients with GC frequently face challenges such as the absence of reliable early diagnostic biomarkers, frequent metastasis, and resistance to standard therapies, all of which contribute to poor clinical outcomes (61–63). Both GPR35a and GPR35b are present in GC, with the mRNA level of GPR35b being significantly higher than that of GPR35a. Given the high expression and transforming activity of GPR35 in GC, these two novel isoforms are likely involved in gastric tumorigenesis (53) (**Table 1**). Furthermore, emerging evidence suggests that GPR35 activation in GC may be regulated through ERR-mediated mechanisms, offering a novel deorphanization and activation pathway (31).

GPR35 facilitates immune evasion in GC by depleting key immune cell populations, such as CD8^+^ T cells and CD4^+^ memory T cells, and by altering immune cells, particularly affecting T cells and macrophages. These alterations contribute to immune suppression and tumor progression (**Figure 1**). Notably, GPR35 is highly expressed in CTSB^+^ and CD68^+^ macrophages and may serve as an early genetic indicator of GC (31). Mechanistically, GPR35 modulates intracellular calcium levels, promotes receptor internalization, and activates ERK1/2 in GC via G_α13_ and G_i/o_ signaling, thereby enhancing GC cell migration, invasion, and remodeling of the tumor microenvironment (52). Additionally, CXCL17 influences the tumor milieu by upregulating CCL20 expression in HGC27 cells. CCL20, in turn, regulates the behavior of fibroblasts, macrophages, and immune cells (64). While CCL20 has tumor-promoting functions, it also recruits dendritic cells and elicits antitumor immune responses under certain condition (65), indicating a context-dependent dual role (**Figure 1**).

### 4.3 GPR35 in colorectal cancer

Colorectal cancer, a prevalent malignancy of the gastrointestinal tract, ranks third in global cancer incidence and is the second leading cause of cancer-related mortality worldwide (1). Despite the availability of chemotherapy and targeted therapies, treatment efficacy is often undermined by drug resistance, adversely affecting patient survival outcomes (66–68). Elevated GPR35 expression has been observed in individuals at increased risk of CRC, and its expression level is significantly correlated with the advanced stage of the tumor and poor prognosis of patients (**Table 2**), implicating the receptor in tumor initiation and progression. However, its direct causal role still requires further verification (69, 70) (**Table 1**). In addition, mRNA expression of GPR35a and GPR35b has been detected in normal intestinal mucosa (69), but there are still differences in the expression distribution and function of GPR35a and GPR35b. For example, in these colon tissues or cells, GPR35b is the predominantly expressed subtype, with its expression level being much higher than that of GPR35a, and GPR35b mRNA serves as a marker for poor prognosis in patients with colon cancer (69). GPR35 and its putative ligand CXCL17 are both highly expressed in CRC tissues and positively correlate with poor clinical outcomes (71). Notably, upregulation of GPR35 and CXCL17 in drug-resistant tumors highlights their role in mediating chemoresistance. Silencing of CXCL17 has been shown to reduce GPR35 expression and inhibit tumor cell proliferation, supporting the therapeutic potential of targeting GPR35–CXCL17 axis (44). *In vitro* studies using GPR35-deficient (*Gpr35^-/-^)* mice in *Apc^min^* and azoxymethane/dextran sodium sulfate (AOM/DSS) models have demonstrated significant reductions in tumor burden, likely due to disrupted NKA-Src signaling and downstream effectors such as ERK1/2 and Akt (26). Furthermore, GPR35 has been shown to promote anchorage-independent growth and malignant transformation through regulation of YAP/TAZ transcriptional coactivators. Its inhibition by the antagonist CID-2745687 suppresses these oncogenic effects (56).

**Table 2:** Expression difference of GPR35 between normal and tumor tissues.

Cancer type	Expression in normal tissue	Expression in tumor (up/down-regulated)	Clinicopathological features	Prognostic value	Refs
HCC	–	Up-regulated	Positively correlated with the presence of liver cirrhosis	High expression of CXCL17 is significantly associated with shorter overall survival (p = 0.015)	(50)
GC	–	Up-regulated	Positively correlated with advanced TNM stage	High expression is significantly correlated with shorter overall survival (OS) (p = 4.30e-08)	(31)
CRC	High	Significantly up-regulated	Positively correlated with lymph node metastasis status	High expression is primarily associated with poor prognosis (P = 0.002 when combined with CEA)	(69)
PDAC	Low	Up-regulated	–	–	(54)

Blocking GPR35 signaling also reduces the secretion of angiogenic factors, including vascular endothelial growth factor (VEGF) and CXCL1, thereby inhibiting tumor angiogenesis and tumor cell proliferation (30) (**Figure 1**). Additional evidence comes from studies on neuroglobin (NGB), which is frequently downregulated in CRC. Overexpression of NGB leads to decreased GPR35 expression, enhanced its degradation, and subsequent inhibition angiogenesis and metastasis (55). Collectively, these findings establish GPR35 as a central regulator of CRC development chemoresistance, and angiogenesis, its potential as a therapeutic target.

### 4.4 GPR35 in pancreatic cancer

Pancreatic ductal adenocarcinoma is among the most lethal malignancies, with a dismal 5-year survival rate of less than 10%, and below 3% for patients (72). Despite advancements in multimodal treatments, including surgery, chemotherapy, radiotherapy, and immunotherapy, clinical outcomes remain poor due to rapid treatment resistance (73–75). The expression of GPR35 is significantly elevated in PDAC tumor tissues, and its expression level shows a positive correlation trend with tumor stage, grade, and resectability, suggesting it may represent a potential biomarker for pancreatic cancer. However, additional clinical studies are required to validate its suitability as an early diagnostic marker (54). The KYN pathway, a known modulator of immune tolerance and tumor growth in PDAC, has been identified as a key activator of GPR35 (76). Silencing GPR35 using siRNA leads to a marked reduction in TGF-β1 expression, a critical mediator of cell dedifferentiation, metastasis, and immune suppression in PDAC (57) (**Figure 1**). The downregulation of GPR35 inhibits AKT phosphorylation at Ser473, thereby compromising the protein stability of its key downstream effector HIF-1α. Inactivation of this signaling axis ultimately suppresses cancer cell proliferation and promotes apoptosis. Additionally, GPR35 silencing disrupts autophagic flux by altering the expression of critical autophagy-regulating proteins, such as LC3B lipidation and p62 accumulation, and concurrently induces G0/G1 cell cycle arrest. These combined effects significantly reduce the viability of pancreatic cancer cells under stress conditions, including hypoxia and nutrient deprivation. This revision description makes explicit that “GPR35 downregulation” leads to the inactivation of the AKT/HIF-1α signaling pathway, which in turn produces two synergistic anti-tumor outcomes: inhibition of proliferation and promotion of apoptosis (58).

In addition to KP (kynurenine pathway), CXCL17 plays a crucial role in PDAC development. CXCL17 is upregulated in intraductal papillary mucinous adenomas, promoting immune cell infiltration by recruiting immature medullary dendritic cells. Its downregulation in intraductal papillary mucinous carcinomas may signal the onset of immune escape and tumor progression (59).

## 5 GPR35 plays as a key regulator of diverse metabolic pathways

Since the identification of KYNA as an endogenous agonist of GPR35, the critical regulatory role of this receptor in metabolic processes has become increasingly evident. GPR35 is highly expressed in white adipose tissue and the liver, where it regulates adipocyte metabolism, thermogenesis, hepatic gluconeogenesis, cholesterol homeostasis, and bile acids (BAs) biosynthesis (77). Additionally, GPR35 activation promotes intestinal secretion of glucagon-like peptide-1 (GLP-1), thereby mediating the incretin effect and contributing to appetite regulation. GPR35 signaling also influences gut microbiota composition and enhances colonic epithelial cell sensitivity to *enterotoxigenic Bacteroides fragilis* (ETBF), exacerbating intestinal inflammation (78). Given its multifaceted roles in improving insulin sensitivity, glycemic control, lipid metabolism, body weight regulation, and inflammatory responses, GPR35 has emerged as a promising therapeutic target for metabolic disorders such as type 2 diabetes mellitus (T2DM), obesity, and NAFLD (79).

### 5.1 GPR35 and glucose metabolism

**Figure 2:**
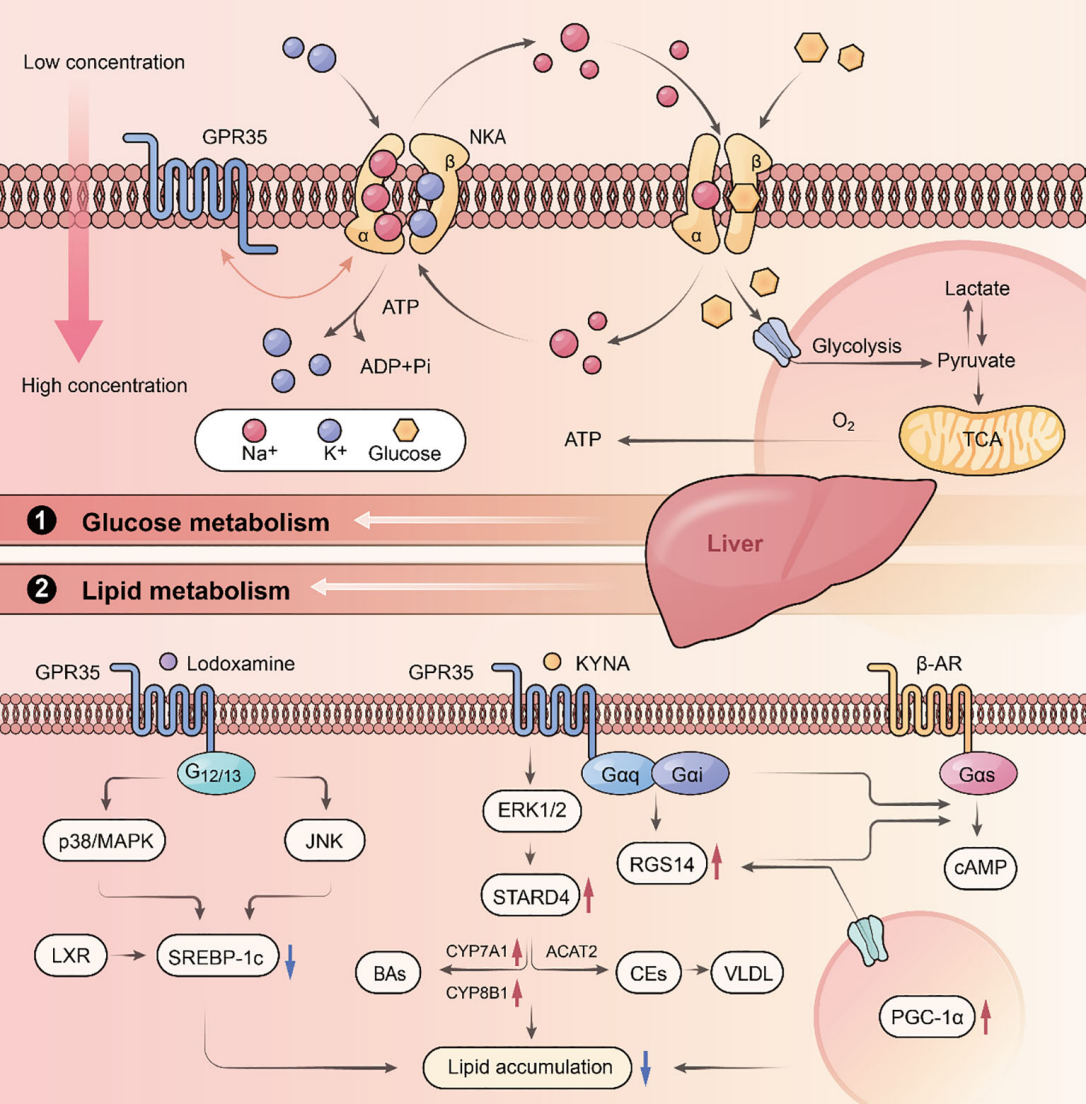
Impact of GPR35 on glucose and lipid metabolic pathways. (1) In glucose metabolism, GPR35 interacts with the α-subunit of the NKA, promoting glycolysis, wherein glucose is converted to pyruvate. Under aerobic conditions, pyruvate enters the mitochondria to fuel ATP production. (2) In lipid metabolism, the GPR35 agonist lodoxamide inhibits hepatic lipid accumulation in a concentration-dependent manner through activation of the p38 MAPK and JNK signaling pathways. KYNA-activated GPR35 signaling induces the expression of steroidogenic acute regulatory protein-related lipid transfer domain-containing 4 (STARD4) via ERK1/2 phosphorylation, thereby promoting cholesterol esterification and BAs synthesis in hepatocytes, as well as adipose tissue remodeling through enhanced thermogenesis. p38/Mitogen-activated protein kinase: p38/MAPK, c-Jun N-terminal kinase: JNK, Liver X receptor: LXR, Bile acids: Bas, StAR related lipid transfer domain containing 4: STARD4, Sterol Reg-Ulatory Element Binding Protein-1c: SREBP-1c, Regulator of G-protein signaling 14: RGS14.

Carbohydrates are essential energy sources that support cellular homeostasis and physiological function. Dysregulation of glucose metabolism is implicated in a range of diseases, including metabolic disorders, cardiovascular disease, and cancer (80). Recent studies have revealed that GPR35 influences glucose metabolism through multiple signaling pathways. GPR35 interacts with the α-subunit of the NKA, enhancing its ion transport function and promoting Src kinase activation (**Figure 2**). This cascade increases cellular glucose demand and accelerates glycolysis, facilitating energy production and supporting tumor cell proliferation (26) (**Table 3**). Additionally, lysophosphatidylcholine 17:0, a lipid metabolite with an odd-carbon chain, upregulates GPR35 expression and has been shown to reduce blood glucose levels and improve insulin sensitivity in high-fat diet-fed mice by enhancing GLP-1 secretion and insulin release (78) (**Table 3**). Gpr35 knockout mice exhibit progressive weight gain and glucose intolerance, indicating that GPR35 deletion disrupts glycolysis-dependent energy metabolism and impairs overall metabolic health (21).

**Table 3:** Role of GPR35 in metabolic regulation.

Metabolism	Metabolic pathway	Refs
Glucose metabolism	Promotes glycolytic processes, glucose transport, cell proliferation	(26)
	Improves glycemic control and insulin resistance	(78)
	Regulates adipose tissue energy homeostasis and inflammation	(21)
Lipid metabolism	Inhibits lipid accumulation	(25, 81)
	Enhances BAs synthesis and ameliorates MAFLD	(77)
	Promotes adipocyte thermogenesis and β-adrenergic signaling	(21)
	Regulates lipid accumulation, inflammation and metabolism-related factor expression	(23)
Tryptophan metabolism	KYNA triggers Ca²^+^ mobilization and inositol phosphate production	(41)
	Reduces acetic acid-induced writhing responses in mice	(45)
	Triggers monocyte adhesion and activation	(86)
	Suppresses inflammatory cytokines and alleviates endometritis	(85)
	Participates in mitochondrial remodeling and provides ischemic protection	(87)
Gut microbe and metabolism	Regulates gut microbial metabolite balance	(79, 93)
	Drives Th17 immune responses, exacerbating experimental encephalitis	(99)
	Maintains intestinal homeostasis	(94, 96)
	GPR35 plays a role in colitis.	(95, 97)
	Promotes epithelial repair by *Lacticaseibacillus paracasei*-derived collagen peptides	(98)

### 5.2 GPR35 and lipid metabolism

Lipid metabolism is essential for maintaining cellular energy balance, membrane integrity, hormone synthesis, and intracellular signaling. Dysregulation of lipid metabolism contributes to metabolic diseases such as obesity, NAFLD, and nonalcoholic steatohepatitis (NASH) (81). GPR35 plays a crucial role in modulating hepatic lipid metabolism and has been implicated in the pathogenesis of these disorders. In NASH models, enhances the expression of STARD4 and upregulates the key enzymes CYP7A1 and CYP8B1, which catalyze the conversion of cholesterol into BAs (**Table 3**). Simultaneously, GPR35 increases the expression of acetyl-coenzyme A cholesterol acyltransferase 2, facilitating cholesterol esterification, thereby reducing free cholesterol levels and preventing lipid accumulation in hepatocytes. This action attenuates lipotoxicity and mitigates inflammation and fibrosis in the liver (77) (**Figure 2**).

GPR35 also modulates phospholipid homeostasis, inflammation, and hepatocyte repair by regulating the expression of lipid metabolism-related genes, playing a protective role in liver function and disease progression (23). The GPR35 agonist lodoxamide has been shown to reduce hepatic lipid accumulation via the p38 MAPK and JNK pathways, reinforcing the receptor’s hepatoprotective effects (25). Furthermore, GPR35 suppresses liver X receptor (LXR)-mediated lipid accumulation and downregulates lipogenic gene expression, thereby limiting lipid synthesis and storage in hepatocytes (81).

Beyond the liver, GPR35 is also involved in systemic lipid metabolism. It promotes the expression of thermogenic and energy expenditure genes such as uncoupling protein 1, PGC-1α, and PR domain-containing 16. It also enhances β-adrenergic receptor signaling in adipocytes, partly through the upregulation of regulator of G protein signaling 14 (21) (**Figure 2**). GPR35 contributes to exercise-induced browning of white adipose tissue, a process critical for energy homeostasis. Notably, *Gpr35^−^/^−^* mice exhibit weight gain and glucose intolerance, further underscoring the receptor’s central role in lipid and glucose metabolism (21). Collectively, these findings establish GPR35 as a key regulator of lipid metabolism and position it as a promising therapeutic target for metabolic disorders characterized by lipid dysregulation.

### 5.3 GPR35 and tryptophan metabolism

**Figure 3:**
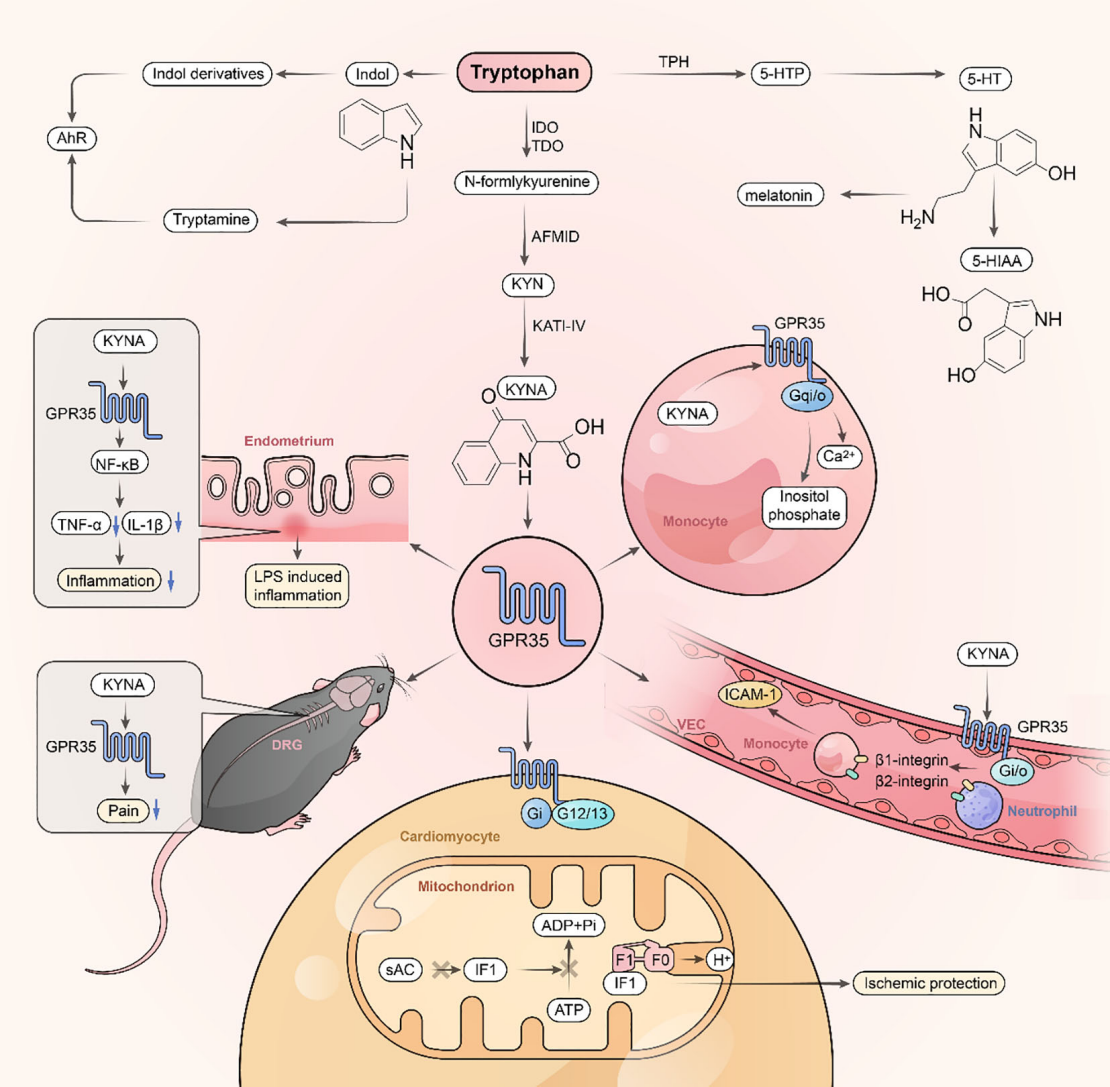
GPR35 and tryptophan metabolism. KYNA orchestrates diverse biological effects through activation of the GPR35 receptor. It suppresses inflammatory signaling by engaging the GPR35/NF-κB axis and alleviates acetic acid-induced visceral pain in mice. Mechanistically, KYNA triggers Gi and G12/13-coupled pathways to maintain mitochondrial bioenergetic homeostasis, promotes leukocyte-endothelial adhesion, and induces intracellular calcium flux, phosphoinositide biosynthesis, and receptor internalization, highlighting its role in immune regulation and cellular energy balance. Tumor necrosis factor-alpha: TNF-α, Arylformamidase: AFMID, Intercellular cell adhesion molecule-1: ICAM-1.

Amino acid metabolism is fundamental to protein synthesis and the generation of bioactive molecules essential for maintaining physiological homeostasis. Disruptions in amino acid metabolism are associated with a range of disorders, including malnutrition, hepatic and renal dysfunction, and systemic inflammatory conditions. Among amino acids, tryptophan plays a particularly critical role in regulating inflammation and maintaining intestinal mucosal integrity (82). More than 95% of dietary tryptophan is metabolized through the KP, producing intermediates such as N-formyl KYN and KYN (83). KYN serves diverse functions in immune regulation, neuronal health, and intestinal homeostasis (84). KYNA, a downstream metabolite of KYN, modulates immune responses and exerts anti-inflammatory effects by inhibiting the production of tumor necrosis factor-alpha (TNF-α) and interleukin-1 beta, as well as suppressing nuclear factor-kappa B (NF-κB) activation in lipopolysaccharide-stimulated mouse endometrial epithelial cells, an effect mediated via GPR35 activation (85) (**Figure 3**). KYNA also binds to GPR35 on human peripheral monocytes, triggering adhesion and cellular activation, thereby contributing to immune surveillance and response modulation (86) (**Table 3**). Beyond its immunomodulatory role, KYNA acts as an N-methyl-D-aspartate receptor antagonist, reducing neuronal hyperexcitability, oxidative stress, and neuroinflammation, thereby providing neuroprotection.

GPR35 is activated by micromolar concentrations of KYNA and plays a pivotal role in intestinal wound repair, an essential process for maintaining gut barrier function. Inhibition of GPR35 signaling disrupts KYNA metabolism, leading to impaired repair of intestinal mucosal damage (27). Furthermore, KYNA–GPR35 interaction activates Gi- and G12/13-coupled signaling pathways and facilitates binding to ATP synthase inhibitory factor subunit 1 on the outer mitochondrial membrane (**Figure 3**). This interaction promotes ATP synthase dimerization, preserving mitochondrial ATP during ischemic stress and protecting against cardiac injury (87).

### 5.4 GPR35 and microbial metabolism

**Figure 4:**
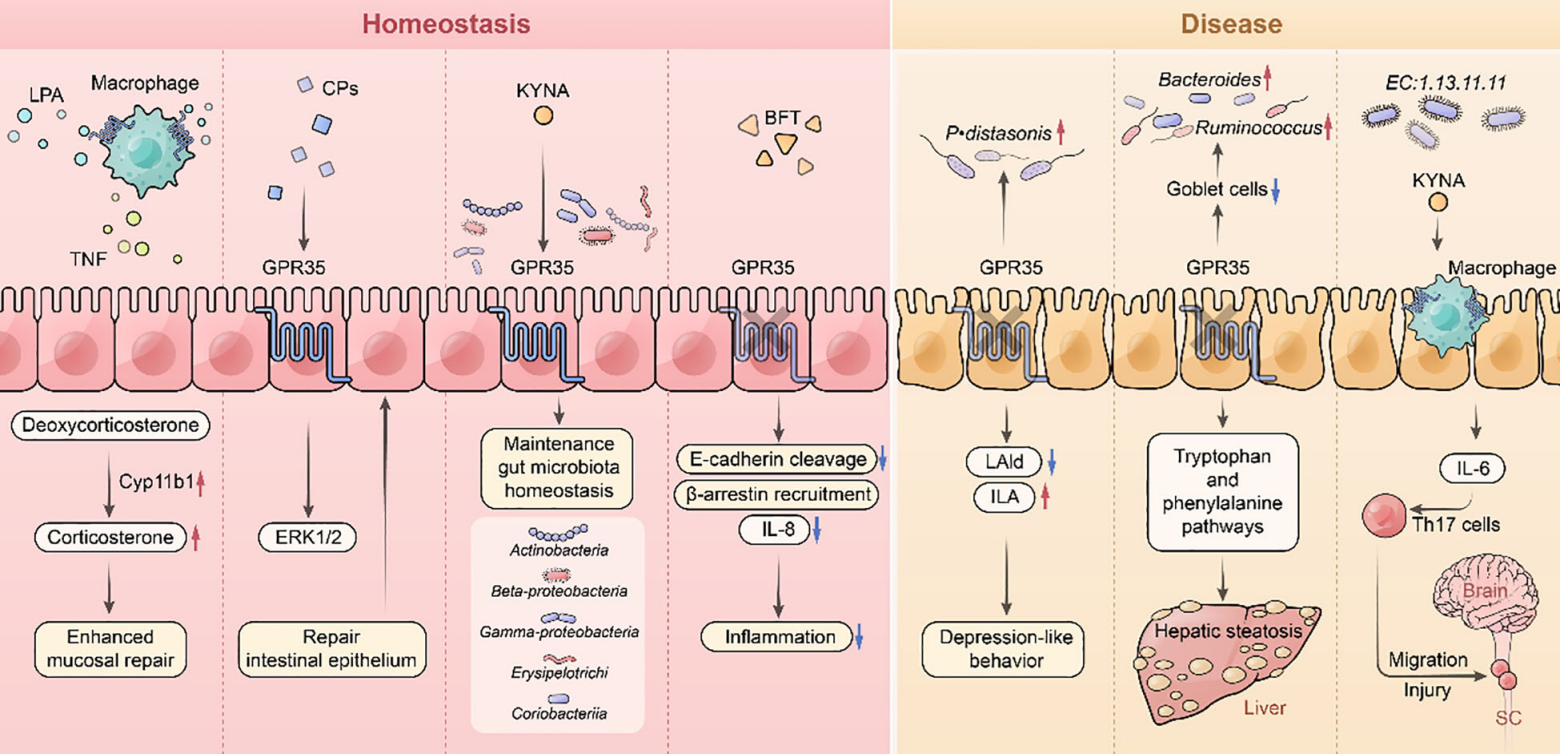
GPR35 and the gut microbiome. GPR35 signaling in CX3CR1^+^ macrophages mediates LPA-induced TNF and Cyp11b1 expression, leading to elevated corticosterone production that sustains intestinal homeostasis. GPR35 also activates ERK1/2 to promote epithelial repair and mediates KYNA sensing to ameliorate DSS-induced colitis. GPR35 deficiency compromises intestinal barrier integrity, exacerbates BFT-induced inflammation, induces dysbiosis, and alters microbial-derived neuroactive metabolites-contributing to depressive behaviors. This deficiency also reduces goblet cell populations and remodels microbial tryptophan/phenylalanine metabolism, potentiating hepatic steatosis. Microbiota-derived KYNA recruits GPR35^+^ macrophages to amplify Th17 responses, thereby linking intestinal inflammation to encephalitis. Lysophosphatidic acid: LPA, Indole-3-carboxaldehyde: IAld, Indole-3-lactate: ILA.

The gut microbiome is a key regulator of host metabolic homeostasis and is implicated in the development of various metabolic disorders, including obesity and NAFLD (88). Microbial diversity and compositional balance are essential to health and are shaped by dietary patterns (89), host genetics, and pharmacological interventions (90). Disruption of the gut microbiota is strongly associated with the onset and progression of metabolic and inflammatory diseases (91). GPR35 functions as a molecular sensor for microbial composition and microbial-derived metabolites (92). In *Gpr35^-/-^* mice, an increased abundance of *Parabacteroidoides distasonis* correlates with decreased serum levels of indole-3-carboxaldehyde (IAld) and elevated levels of indole-3-lactic acid (ILA) (93). IAld enhances neurite outgrowth and synaptic function, particularly in the nucleus ambiguus, whereas ILA exhibits inhibitory effects on neuronal activity (93) (**Figure 4**). These findings are consistent with human studies, where reduced IAld levels and increased *P. distasonis* abundance have been observed in patients with depression, suggesting a potential role for GPR35 in neuroimmune regulation and mood disorders (93).

In the gastrointestinal tract, GPR35 may contribute to the protection of intestinal integrity and microbial homeostasis. For example, GPR35 is thought to protect against ulcerative colitis by monitoring KYNA levels and maintaining mucosal barrier function (94). GPR35 also mediates host responses to ETBF. Its inhibition reduces epithelial responses to *B. fragilis* toxin (BFT), including E-cadherin cleavage, β-arrestin recruitment, and IL-8 secretion, key events that exacerbate colitis (95) (**Table 3**). During intestinal inflammation, the endogenous GPR35 ligand LPA stimulates TNF production in macrophages. GPR35 deficiency leads to impaired TNF synthesis and reduced corticosterone levels, thereby aggravating colitis severity (96). These observations further implicate GPR35 in mucosal immune regulation. Recent studies support GPR35’s therapeutic potential in colitis. Ethanol extract of *Limonium bicolor* has been shown to restore microbial balance in mice with ETBF-induced colitis by reducing Proteobacteria and increasing probiotic genera such as *Lactobacillus* and *Blautia* (97). Additionally, collagen peptides derived from *Lactobacillus paracasei* promote intestinal epithelial repair via GPR35-mediated ERK1/2 signaling (98) (**Figure 4**).

Beyond colitis, KYNA-GPR35 interaction facilitates macrophage recruitment and promotes T-helper 17 (Th17) cell accumulation in the small intestine, contributing to experimental autoimmune encephalomyelitis pathogenesis (99). GPR35 deficiency disrupts microbiota composition, increasing the relative abundance of genera such as *Bacteroides* and *Ruminococcus*. In particular, *Ruminococcus gnavus*, when combined with a high-fat diet, promotes obesity and hepatic steatosis in mice (23). Collectively, these findings establish GPR35 as a vital molecular link between the gut microbiome, immune regulation, and host metabolism.

## 6 Targeting GPR35 to modulate metabolic reprogramming: a promising therapeutic strategy for cancer

Metabolic reprogramming is a hallmark of cancer, enabling tumor cells to sustain proliferation, resist apoptosis, and adapt to oxidative stress by altering glycolysis, oxidative phosphorylation, amino acid utilization, lipid metabolism, and nucleotide biosynthesis (15, 100). Digestive system cancers, characterized by high metabolic demand, frequently display dysregulated metabolic pathways. As previously discussed, GPR35 is highly expressed in various cancers and governs key metabolic processes, particularly glucose and lipid metabolism, during tumorigenesis. Due to its favorable druggable structure and the feasibility of designing high-affinity agonists and inhibitors, pharmacological modulation of GPR35 represents a promising strategy for treating digestive and other cancers. In this section, we examine the mechanisms by which GPR35 drives tumor development via metabolic reprogramming and highlight potential avenues for therapeutic intervention.

### 6.1 Targeting GPR35 to reprogram glucose metabolism in cancer

Rapid proliferation of cancer cells is largely dependent on aerobic glycolysis, a phenomenon known as the Warburg effect, despite its lower ATP yield compared to mitochondrial oxidative phosphorylation (101). Tumors display increased glucose uptake and conversion to lactate, facilitating biosynthesis and energy production (102). Hallmarks of this metabolic phenotype include overexpression of glucose transporters such as GLUT1 and GLUT3, which enhance glucose influx (103). GPR35 contributes to tumor cell metabolism by interacting with the α-subunit of the Na^+^/K^+^-ATPase, activating Src kinase signaling, and promoting the secretion of the neoangiogenic factors such as VEGF and CXCL1. This cascade supports angiogenesis and tissue remodeling, facilitating tumor growth (26) (**Table 4**). GPR35-mediated activation of Src also influences downstream effectors including ERK1/2 and Akt, modulating cell proliferation and survival (104).

**Table 4:** Mechanistic evidence indicates that GPR35 drives cancer progression by reprogramming cellular metabolism.

Cancer	Metabolism	Molecular mechanism	Refs
CRC	Glucose metabolism	Interacts with NKA to drive glycolysis and tumorigenesis	(26)
	Lipid metabolism	Promotes BAs synthesis, inhibits anti-tumor immunity	(105)
	Amino acid metabolism	Aggravates immunosuppression, fueling CRC liver metastasis	(106)
	Gut microbial metabolism	IL-17/NF-κB drives distal colon tumorigenesis	(107)
HCC	Glucose metabolism	Promotes epithelial-mesenchymal transition and drives HCC progression	(108)
	Lipid metabolism	Exacerbates steatohepatitis and HCC initiation	(77)
	Amino acid metabolism	KYN/AHR blockade boosts anti-Tumor T Cells in HCC	(109)
GC	Glucose metabolism	Modulates IFN/MAPK to promote proliferation, apoptosis, migration and invasion	(110)
	Lipid metabolism	Promotes gastric epithelial cells growth	(111)
	Amino acid metabolism	Reduces CD4^+^ T and CD8^+^ T cell infiltration in GCs	(112)
	Gut microbial metabolism	Disrupts gut microbiota balance and promotes gastric cancer development	(113)
Pancreatic cancer	Lipid metabolism	BAs activates FXR in the pancreas and reduces pancreatic cancer	(114)
	Amino acid metabolism	KYNA/GPR35 are potential anti-cancer targets	(57)

By regulating NKA activity, GPR35 indirectly maintains intracellular calcium homeostasis, which is critical for signaling pathways governing cell cycle progression, apoptosis, and differentiation. Through this mechanism, GPR35 supports glucose uptake and enhances glycolytic flux, potentially via modulation of glycolytic enzyme activity and ATP production (26). GPR35 has also been shown to coordinate aerobic glycolysis and oxidative phosphorylation in macrophages and intestinal epithelial cells, promoting energy homeostasis through NKA-mediated enhancement of both metabolic pathways (26). Given the dual role of NKA in both ion transport and signal transduction, its dysregulation in cancer is notable. NKA functions as a dual regulator: in normal tissues, it supports cell proliferation (115), while in cancer cells, its modulation affects signaling molecules involved in invasion and metastasis, such as Rac/Cdc42, profilin, ERK1/2, and P70S6K (116).

Increased NKA activity is associated with enhanced glycolytic metabolism in tumor cells, supporting rapid growth. Conversely, NKA inhibition has therapeutic potential in both cardiovascular and neoplastic diseases (117). The ATPase Na^+^/K^+^ transporting subunit alpha 1 (ATP1A1), a key NKA component, regulates ionic balance and cell volume, and contributes to gastric cancer progression by modulating interferon (IFN) and MAPK signaling. High ATP1A1 expression is linked to poor prognosis in GC patients (110) (**Table 4**). Specific NKA inhibitors, such as digoxin and ouabain, exhibit antitumor effects. Digoxin increases intracellular Ca²^+^, activates stress pathways, and induces cell cycle arrest and apoptosis. It also inhibits HIF-1α, NKA, and NF-κB, enhancing its anticancer properties (118). Ouabain exerts cytotoxic effects by increasing intracellular Na^+^ and Ca²^+^, inhibiting proliferation, and inducing apoptosis and cell cycle arrest (119). *In vivo* studies confirm that NKA inhibition reduces tumor burden in HCC by suppressing angiogenic factors, pro-survival signaling, and metastatic potential (108). Together, these findings support a therapeutic model in which targeting GPR35 and associated glycolytic signaling, particularly through NKA modulation, may offer novel treatment avenues for metabolic reprogramming in cancer.

### 6.2 Targeting GPR35 to reprogram lipid metabolism in cancer

Cancer cells undergo extensive lipid metabolic reprogramming to meet elevated demands for energy, membrane synthesis, and signaling molecules essential for rapid proliferation and survival (120). This metabolic shift is a hallmark of cancer, enabling tumor cells to resist apoptosis and adapt to the nutrient-depleted, hypoxic tumor microenvironment (121). Compared to normal cells, cancer cells exhibit enhanced *de novo* lipogenesis, increased fatty acid uptake, and altered β-oxidation pathways (120). GPR35 activation by the agonist lodoxamide has been shown to inhibit hepatic lipid accumulation (25). In the context of NASH, GPR35 reduces steatohepatitis severity by inducing STARD4 expression and promoting cholesterol conversion into BAs, thereby attenuating lipotoxicity (77) (**Table 4**). Dysregulated BA metabolism has been associated with elevated risk of digestive cancers, influencing disease progression via alterations in gut microbiota composition, nuclear receptor farnesoid X receptor (FXR) signaling, and immune modulation (122).

Studies in patient samples and animal models reveal that BA pool size and the expression of key synthases increase with STARD1 upregulation. High-cholesterol diets elevate BA levels, contributing to HCC progression by influencing gene expression and inflammatory signaling pathways (123). Furthermore, secondary BAs, produced via microbial transformation of primary Bas, are implicated in colorectal cancer (124). Notably, deoxycholic acid, a secondary BA elevated in CRC, suppresses antitumor immunity by enhancing plasma membrane Ca²^+^ ATPase activity and dampening the Ca²^+^-NFAT2 pathway, reducing IFN-γ and TNF-α expression and promoting tumor growth (105) (**Table 4**). In PDAC, elevated FXR expression correlates with lymph node metastasis and poor prognosis (125). BA–FXR signaling inhibits autophagy by downregulating Atg7 and LAMP-2 and promotes cytokine expression (e.g., TNF-α, IL-6, IL-10, TGF-β), modulating immune responses and delaying PDAC progression (114) (**Table 4**). In GC, BAs activate the IL-6/JAK1/STAT3 pathway, alter gastric pH, promote epithelial proliferation and inflammation, collectively driving tumorigenesis (111). Given GPR35’s regulatory role in BA synthesis and lipid metabolism, pharmacological modulation of GPR35 activity may provide a promising strategy to disrupt lipid metabolic dependencies in digestive cancers.

### 6.3 Targeting GPR35 to reprogram amino acid metabolism in cancer

Amino acids are essential intermediates for biosynthesis and serve as key energy sources in cancer cells, supporting the synthesis of proteins, nucleotides, and redox molecules (126). Among tryptophan metabolites, 5-HIAA is a GPR35 agonist that plays an important role in neutrophil activation (20). During inflammation, GPR35 is upregulated in activated neutrophils and eosinophils, enhancing their migratory capacity. Concurrently, platelets and mast cells produce 5-HIAA, which promotes transendothelial migration of these immune cells (127). However, excessive neutrophil infiltration may foster chronic inflammation and colorectal tissue damage via reactive oxygen species, increasing the risk of genetic instability and tumor development (128, 129). Moreover, neutrophils can transfer lipids to tumor cells, thereby supporting tumor proliferation and survival (130).

KYNA, another key tryptophan-derived metabolite, plays a critical role in intestinal homeostasis. In chemotherapy-induced intestinal injury, the tryptophan-KYNA axis is upregulated. Both GPR35 and the aryl hydrocarbon receptor (AHR) function as sensors of KYN metabolism, modulating inflammation and preserving mucosal integrity through KYNA gradient sensing (27), the KYNA-GPR35 axis has also been identified as a “molecular switch” that regulates host appetite (131). Notably, recent studies have shown that “superfoods” containing ligands for the AhR and GPR35 receptors can modulate intestinal immune and inflammatory responses, holding significant importance for preventing colorectal cancer in IBD patients (132). Indoleamine 2,3-dioxygenase (IDO) and tryptophan 2,3-dioxygenase (TDO) are overexpressed in various cancers and catalyze tryptophan degradation to KYN, which is further converted to KYNA by KYN aminotransferases (133). Alterations in IDO activity are potential biomarkers for CRC therapy response (134), with CRC patients exhibiting decreased serum tryptophan and elevated KYN levels (135). Endothelial IDO expression in CRC correlates with recurrence and hepatic metastases and is associated with reduced CD3^+^ T cell infiltration (106) (**Table 4**). Pharmacological inhibition of GPR35 using TMER1i enhances antitumor immune responses by disrupting Hippo-YAP signaling in T regulatory and cytotoxic T cells, counteracting IDO1-driven immunosuppression in the tumor microenvironment (136). Elevated serum KYN levels in HCC patients predict poor prognosis (137), and TDO promotes HCC cell proliferation and invasion (138). Inhibition of TDO reduces KYN-AHR signaling and enhances T cell responses in HCC (109).

In gastric cancer, elevated tryptophan, tyrosine, and phenylalanine levels in gastric fluid are associated with increased IDO expression and a shift toward immunosuppressive regulatory T cells (Tregs) populations (112) (**Table 4**). In pancreatic adenocarcinoma, IDO overexpression predicts poor outcomes and contributes to NK cell dysfunction, which can be alleviated by IDO inhibitors (139). Beyond classical amino acid metabolism, GPR35 regulates osmolyte levels critical for tumor cell survival. In HepG2 cells, GPR35 silencing reduces intracellular concentrations of glycerophosphocholine, glycerophosphoethanolamine, and proline betaine—osmolytes that counteract cellular stress, highlighting GPR35’s role in both nutrient acquisition and stress adaptation (140). Together, these findings underscore GPR35’s dual function in shaping inflammatory responses and reprogramming tumor metabolism, making it a promising target for therapeutic intervention in amino acid metabolism-driven cancers.

Although current research on GPR35-mediated metabolic regulation often focuses on isolated pathways, emerging evidence suggests its potential role as a central integrator that coordinates metabolic networks. Unlike receptors that exert indirect effects, GPR35 demonstrates the potential for “hard-wired” control, enabling coordination of glucose, lipid, and amino acid metabolism. Compelling evidence stems from its direct interaction with the NKA. As the primary engine maintaining membrane potential and sodium gradients, the sodium-potassium pump’s activity is a key driver for sodium-glucose cotransporters (SGLTs) in glucose uptake. This finding positioning suggests that GPR35 may be can simultaneously establish the foundation for glucose metabolism. Furthermore, hepatic GPR35 is indispensable for systemic lipid homeostasis, as its deletion disrupts lipid balance. Moreover, an elegant intrinsic sensing loop exists between GPR35 and its endogenous ligand, KYNA. Since KYNA is a direct product of tryptophan metabolism, the status of amino acid metabolism can directly influence GPR35 activation. This relationship forms a feedforward/feedback circuit, positioning GPR35 as a sensor of intracellular nutrient status capable of orchestrating broad metabolic reprogramming.

### 6.4 Targeting GPR35 to reprogram microbial metabolites in cancer

The gut microbiota plays an integral role in immune homeostasis, and its dysregulation is linked to chronic inflammation and cancer development, particularly in colorectal cancer (141, 142). GPR35 is highly expressed in colonic epithelial cells and acts as a molecular bridge between microbial metabolites and host signaling pathways, contributing to intestinal immune regulation (96). In mouse models, GPR35 mediates host responses to ETBF by sensing BFT and initiating downstream signaling cascades, including β-arrestin recruitment, E-cadherin cleavage, and IL-8 secretion that promote inflammation and epithelial barrier disruption (95). ETBF-host interactions drive chronic inflammation, a known risk factor for CRC development (143).

ETBF is significantly enriched in the colonic mucosa and feces of CRC patients compared to controls (144). In *Apc^Min/+^* mice, ETBF induces distal colon tumors via IL-17 and NF-κB signaling pathways (107) (**Table 4**). ETBF also contributes to gastric carcinogenesis by disrupting intercellular junctions and promoting M1 macrophage transmigration across the epithelium, leading to inflammation and neoplasia (113) (**Table 4**). Given its role in regulating host responses to microbial signals, GPR35 represents a key node in the inflammation-microbiome-cancer axis. Therapeutically targeting GPR35 to modulate gut microbial interactions could offer a novel approach to preventing or treating digestive system cancers.

## 7 A comprehensive view of GPR35 in digestive system cancers: current efforts and existing challenges

### 7.1 Species-specific challenges

A long-standing central issue in GPR35 research stems from the significant species selectivity of its ligand pharmacology between humans and rodents. The ligand-binding pocket of human GPR35 features an “upper” region rich in positively charged residues and a neutral “lower” region, whereas the rat orthologue presents the opposite configuration–a neutral upper region and a positively charged lower region. For instance, lodoxamide was the first agonist reported to exhibit high and nearly equal potency at both human and rat GPR35, yet it is virtually ineffective at the mouse GPR35. Paradoxically, *in vivo* studies in mice have shown that lodoxamide exerts a GPR35-dependent anti-liver fibrosis effect through some unknown mechanism, highlighting its complex species-dependent differences (145). These structural differences cause negatively charged ligands to adopt distinct binding orientations, which partly explains why certain ligands are highly potent at human GPR35 but largely ineffective in rats/mice (146). Consequently, results from existing animal studies are difficult to extrapolate directly to humans, significantly impeding the clinical translation of preclinical data.

### 7.2 Targeting GPR35 in digestive system cancers: current explorations

In recent years, the role of GPR35 in tumor development has gained increasing attention. Its functional regulation can occur either through activation by endogenous ligands or via its intrinsic activity independent of ligands.

In terms of ligand-dependent activation, several endogenous ligands have been identified. The endogenous agonist KYNA, a tryptophan metabolite, not only functions in the central nervous system and possesses antioxidant properties (21), but also shows increased expression in colonic mucosa after colon cancer chemotherapy. It can directly activate epithelial GPR35, induce receptor internalization, and promote ERK1/2 phosphorylation, thereby accelerating epithelial cell migration and injury repair. This suggests a protective role of the KYNA-GPR35 axis in chemotherapy-induced intestinal toxicity. However, this mechanism doesn’t directly participate in cancer progression or drug resistance itself, and its effects may vary depending on cancer type, stage, and microenvironment (27). Another ligand, CXCL17, plays a central role in various malignancies, associated with tumor development, invasion and metastasis (28). In CRC, the CXCL17-GPR35 axis promotes tumor proliferation, migration, invasion and chemotherapy resistance by activating the IL-17 signaling pathway, thereby driving malignant progression (44). In GC, although CXCL17 expression gradually increases in precancerous lesions but significantly decreases in gastric cancer tissues, GPR35 remains consistently highly expressed, suggesting that CXCL17 loss and GPR35 activation may jointly promote gastric cancer progression by upregulating CCL20 to reshape the immune microenvironment (52). Additionally, in HCC, CXCL17 has also been found associated with unfavorable CD4^+^ T cell and CD68^+^ macrophage infiltration (50). On the other hand, GPR35 can also participate in tumor progression through its constitutive activation independent of ligands. In the tumor microenvironment, macrophage GPR35 promotes VEGF, CXCL-1 and MMPs expression through the NKA-Src signaling axis, thereby driving angiogenesis and matrix remodeling. Using the selective inhibitory peptide g35i2 or conditional knockout of this receptor significantly inhibits tumor growth (30). In CRC cells, GPR35 inhibits YAP/TAZ phosphorylation, thereby enhancing YAP/TAZ transcriptional activity and promoting anchorage-independent growth of cancer cells, while small molecule inhibitors CID-2745687 and ML145 can effectively block this pathway (56). In GC, GPR35 knockdown not only inhibits cancer cell proliferation and migration and promotes apoptosis, but also blocks macrophage polarization to M2 phenotype, suggesting its role as an immune regulation node and potential therapeutic target (31). In PDAC, GPR35 promotes tumor proliferation, metabolic reprogramming and metastasis by activating AKT, stabilizing HIF-1α and regulating autophagy (58).

In summary, GPR35 has complex and context-dependent mechanisms in cancer. It can mediate either protective or cancer-promoting effects through different ligands in specific cancer types, and can also regulate multiple oncogenic signaling pathways through its intrinsic activity independent of ligands, making it a potential therapeutic target across multiple cancers.

### 7.3 The molecular landscape of GPR35-mediated cancer progression remains incomplete

The molecular landscape driving cancer progression through GPR35 constitutes a complex and not yet fully connected dynamic network. The incompleteness of our understanding stems not only from gaps in individual signaling pathways, but more importantly, from an insufficient grasp of its context-dependent regulatory logic.

Currently, several molecules have been proposed as endogenous ligands for GPR35; However, their authenticity under physiological conditions and their pathological relevance remain debated. These ligands exhibit significant potency differences across species, and the precise binding sites for most ligands are still unclarified. For instance, KYNA shows significantly higher activation potency for rodent GPR35 compared to the human receptor, demonstrating strong species selectivity (41). At the signaling mechanism level, GPR35 primarily transduces signals by coupling with Gα12/13 and Gαi/o classes of G proteins (147). Upon agonist activation, GPR35 can recruit β-arrestin, leading to receptor internalization and desensitization (148, 149). Furthermore, GPR35 can interact with Na^+^/K^+^-ATPase, trans-activating Src kinase and driving downstream pro-survival and proliferative signals (26). Within the tumor microenvironment, GPR35 can promote macrophage polarization towards the M2 phenotype and the release of pro-angiogenic factors, thereby indirectly supporting tumor growth and shaping an immunosuppressive microenvironment (30).

Although the aforementioned mechanisms are gradually being uncovered, a core limitation of current research lies in the lack of a systematic explanation for the molecular basis of GPR35-mediated cancer progression. Specifically, we still do not understand which mechanisms—such as specific ligands, receptor isoforms, or cellular contexts—determine the signaling bias of GPR35 in different cancer types or stages. Secondly, the specific functional division of labor between the two splice isoforms, GPR35a and GPR35b, remains unclear, and their specific interactomes and downstream biological effects urgently need clarification. Ultimately, the greatest challenge facing this field is how to integrate these disparate signaling modules, intrinsic activities, and immunoregulatory functions into a systems-level model capable of predicting cell fate. This would allow for accurately defining the key dependency states driven by GPR35 in tumor progression, providing a theoretical basis and identifying potential therapeutic windows for targeted interventions.

## 8 Conclusion and future perspectives

GPR35, a member of the largest druggable family in the human, plays distinct and multifaceted physiological roles in cancer growth, metastasis, and metabolic regulation. Aberrant GPR35 expression has been consistently associated with poor prognosis across several malignancies, particularly those affecting the digestive system. GPR35 contributes to cancer progression by orchestrating metabolic reprogramming, directly modulating glucose, lipid, amino acid, and microbial metabolite metabolism. Moreover, it influences the tumor microenvironment and immune responses by regulating the production and utilization of metabolic intermediates. This review provides a comprehensive summary of the role of GPR35 in the metabolic regulation of digestive cancers, highlighting how its signaling axis governs tumorigenesis via metabolic reprogramming. The evidence presented supports GPR35 as a compelling therapeutic target, with intervention in its metabolic pathways offering novel opportunities for cancer treatment.

However, translating this promise into clinical reality requires overcoming multi-layered challenges, spanning fundamental understanding to drug development. A primary obstacle lies in our still-fragmentary grasp of GPR35’s molecular mechanisms, which is further complicated by its context-dependent roles across different cancer subtypes and within the tumor microenvironment. While the two key isoforms, GPR35a and GPR35b, are known to exhibit distinct expression patterns and prognostic associations in gastrointestinal malignancies (e.g., GC and CRC), this likely represents only a fraction of a more complex landscape. Future work must move beyond bulk tissue analysis to investigate the dynamic changes and functional heterogeneity of GPR35 across different molecular subtypes of cancer. For instance, it remains unexplored whether GPR35 expression and signaling are enriched in specific malignant cell subpopulations, such as those with stem-like properties, or how its function is modulated by cues from cancer-associated fibroblasts or immune cells within the tumor microenvironment. Current methodological limitations in discretely studying isoform-specific and cell-type-specific functions leave these critical downstream interactomes and signaling networks largely unmapped. Therefore, employing novel technologies like CRISPR-based gene editing, single-cell multi-omics sequencing, and spatial transcriptomics to delineate the precise functions of each isoform within specific cellular contexts will be crucial. This approach will not only decipher GPR35’s core oncogenic mechanisms but also reveal its adaptive roles in tumor-stroma interactions, representing a highly valuable direction for future research.

Furthermore, the pronounced species-dependent differences in GPR35 ligand pharmacology represent an Achilles’ heel for clinical translation. The inverted binding pocket conformation between human and rodent orthologs renders many compounds effective in preclinical models (e.g., zaprinast) poorly translatable to humans. This bottleneck underscores the urgent need for more predictive humanized GPR35 mouse models, which are indispensable for bridging foundational discoveries and clinical trials.

Encouragingly, despite these hurdles, the clinical exploration of GPR35-targeted drugs has gained tangible momentum. Several compounds for different indications have entered the clinical pipeline (**Table 5**): CT-3001, as the first-in-class GPR35 inhibitor, is being closely watched for its efficacy in advanced cancers (153), while KYNA-based formulations and GSK4381406 are pioneering new avenues in scar modulation and gut-selective therapy, respectively (151, 152). These advances not only validate GPR35’s “druggability” but also provide invaluable human data for the field.

**Table 5:** The GPR35 drug development pipeline: current clinical candidates and preclinical innovations.

Stage	Compound/ intervention	Pharmacological action	Indications	Key findings	Refs
Clinical trial	GSK4381406	Agonists	IBD	Phase I ongoing (NCT05999708); Enhance intestinal barrier function and suppress IBD.	(150)
	KYNA	Agonists	Scar	Phase II trial paused (NCT02340325) due to formulation issues; Mechanistic validation in lipid regulation.	(151, 152)
Clinical trial	CT-3001	Antagonists	Advanced malignant solid tumors, CRC, PDAC	Phase I/II ongoing (NCT06598007); Targets GPR35-mediated immunosuppression in TME.	(153)
Preclinical	Pamoic acid	Agonists	Pain in internal organs	Suppressed via Gi/o-ERK1/2/β-arrestin2 axis, offering a novel analgesic approach for gut disorders.	(154)
	GPR35 inverse agonists	Agonists	Gastrointestinal disorders	Strongly linked to inflammatory bowel diseases	(155)
	TCG1001, Zaprinast	Agonist	Osteoporosis	Activates Gi/o and G12/13 signaling, suppressing osteoclast activity.	(156)
	Cromolyn, Zaprinast	Agonist	Visceral pain (colonic hyperalgesia)	Inhibits colonic nociception, suggesting non-opioid analgesic potential.	(157)
	Lodoxamide	Agonist	Hepatic steatosis	Blocks LXR-SREBP-1c pathway to reduce lipid synthesis.	(81)
	Olsalazine	Agonist	IBD	Attenuates colitis via NF-κB/JAK-STAT3 pathway downregulation.	(158)
	CID-2745687	Antagonists	CRC	Suppresses YAP/TAZ activity.	(56)
	ML145	Antagonists	IBD	Prevents epithelial damage and inflammatory responses.	(95)

Deciphering the intricate biology of GPR35 is the cornerstone for unlocking its full therapeutic promise. Achieving this demands a multidisciplinary strategy focused on two fronts: first, to define the molecular determinants of its signaling preference and build integrated, predictive models of its functions; and second, to create advanced, human-relevant animal models that circumvent the confounding issue of species-specific pharmacology. The ultimate goal is to pioneer novel combination therapies that co-target the GPR35 axis alongside established immuno-oncology or metabolic interventions, thereby overcoming treatment resistance and paving the way for more effective outcomes in difficult-to-treat cancers.

## Glossary

ACAT2: Acyl-coenzyme A cholesterol acyltransferase 2
AMPK: AMP-activated protein kinase
Atg7: Autophagy Related 7
AHR: Aromatic hydrocarbon receptor
AOM/DSS: Azoxymethane/dextran sodium sulfate
ATP1A1: ATPase Na^+^/K^+^ transporting subunit alpha 1
ATPIF1: ATP synthase inhibitory factor subunit 1
BAs: Bile acids
BMP4: Bone morphogenetic protein 4
CECs: Colonic epithelial cells
CCL20: Chemokine (C-C motif) ligand 20
CRC: Colorectal cancer
CXCL1: C-X-C motif chemokine ligand 1
CXCL17: C-X-C motif chemokine ligand 17
CYP7A1: Cytochrome P450 family 7 subfamily A member 1
CYP8B1: Cytochrome P450 family 8 subfamily B member 1
DCA: Deoxycholic acid
ETBF: Enterotoxigenic *Bacteroides fragilis*
ERK1/2: Extracellular signal-regulated kinases 1 and 2
ERR: Enhancer release and retargeting
FXR: Farnesoid X receptor
GC: Gastric cancer
GPCR: G protein-coupled receptor
GPR35: G protein-coupled receptor 35
GLUT1: Glucose transporter 1
GLUT3: Glucose transporter 3
GLP-1: Glucagon-like peptide-1
HCC: Hepatocellular carcinoma
HFD: High fat diet
IAld: Indole-3-carboxaldehyde
ILA: Indole-3-lactate
IBD: Inflammatory bowel disease
IDO: Indoleamine 2,3-dioxygenase
IFN: Interferon
IPMA: Intraductal papillary mucinous adenoma
JNK: c-Jun N-terminal kinase
KYN: Kynurenine
KYNA: Kynurenic acid
KP: Kynurenine pathway
Lamp-2: Lysosome-associated membrane protein 2
LKB1: Liver Kinase B1
LPA: Lysophosphatidic acid
LXR: Liver X receptor
MMPs: Matrix Metalloproteinases
NAFLD: Non-alcoholic fatty liver disease
NASH: Non-alcoholic steatohepatitis
NFK: N-formyl kynurenine
NKA: Na⁺/K⁺-ATPase
NGB: Neuroglobin
NMDA: N-methyl-D-aspartic acid
NSCLC: Non-small cell lung cancer
PDAC: Pancreatic ductal adenocarcinoma;
p38/MAPK: p38 Mitogen-activated protein kinase
Pgc-1α: PPARγ
PMCA: Plasma membrane Ca^2+^ ATPase;
RGS14: Regulator of G-protein signaling 14
ROS: Reactive oxygen species
SGLT2: Sodium-glucose transport protein 2
SLC2A1: Solute carrier family 2 member 1
SMAD: Small mother against decapentaplegic
SREBP-1c: Sterol Reg-Ulatory Element Binding Protein-1c
STARD4: STAR-related lipid transfer domain containing 4
SNP: Single-nucleotide polymorphism
TDO: Tryptophan 2,3-dioxygenase
TNF-α: Tumor necrosis factor-alpha
TG: Triglyceride
Tregs: Regulatory T cell
UCP1: Uncoupling protein 1
VEGF: Vascular endothelial growth factor
WHO: World Health Organization
5-HIAA: 5-hydroxyindole acetic acid

## References

[1] Bray F Laversanne M Sung H Ferlay J Siegel RL Soerjomataram I . Global cancer statistics 2022: Globocan estimates of incidence and mortality worldwide for 36 cancers in 185 countries. CA Cancer J Clin. (2024) 74:229–263. doi: 10.3322/caac.21834, PMID: 38572751

[2] Lu L Mullins CS Schafmayer C Zeißig S Linnebacher M . A global assessment of recent trends in gastrointestinal cancer and lifestyle-associated risk factors. Cancer Commun (Lond). (2021) 41:1137–1151. doi: 10.1002/cac2.12220, PMID: 34563100 PMC8626600

[3] He Z Tian T Guo D Wu H Chen Y Zhang Y . Cytoplasmic retention of a nucleocytoplasmic protein tbc1d3 by microtubule network is required for enhanced egfr signaling. PloS One. (2014) 9:e94134. doi: 10.1371/journal.pone.0094134, PMID: 24714105 PMC3979746

[4] Grady WM Yu M Markowitz SD . Epigenetic alterations in the gastrointestinal tract: Current and emerging use for biomarkers of cancer. Gastroenterology. (2021) 160:690–709. doi: 10.1053/j.gastro.2020.09.058, PMID: 33279516 PMC7878343

[5] Arnold M Abnet CC Neale RE Vignat J Giovannucci EL McGlynn KA . Global Burden of 5 major types of gastrointestinal cancer. Gastroenterology. (2020) 159:335–349.e315. doi: 10.1053/j.gastro.2020.02.068, PMID: 32247694 PMC8630546

[6] Siegel RL Giaquinto AN Jemal A . Cancer statistics, 2024. CA Cancer J Clin. (2024) 74:12–49. doi: 10.3322/caac.21820, PMID: 38230766

[7] Zhao L Liu Y Zhang S Wei L Cheng H Wang J . Impacts and mechanisms of metabolic reprogramming of tumor microenvironment for immunotherapy in gastric cancer. Cell Death Dis. (2022) 13:378. doi: 10.1038/s41419-022-04821-w, PMID: 35444235 PMC9021207

[8] Cao LQ Xie Y Fleishman JS Liu X Chen ZS . Hepatocellular carcinoma and lipid metabolism: Novel targets and therapeutic strategies. Cancer Lett. (2024) 597:217061. doi: 10.1016/j.canlet.2024.217061, PMID: 38876384

[9] Li F Si W Xia L Yin D Wei T Tao M . Positive feedback regulation between glycolysis and histone lactylation drives oncogenesis in pancreatic ductal adenocarcinoma. Mol Cancer. (2024) 23:90. doi: 10.1186/s12943-024-02008-9, PMID: 38711083 PMC11071201

[10] Li J Pan J Wang L Ji G Dang Y . Colorectal cancer: Pathogenesis and targeted therapy. MedComm (2020). (2025) 6:e70127. doi: 10.1002/mco2.70127, PMID: 40060193 PMC11885891

[11] Luo Z Eichinger KM Zhang A Li S . Targeting cancer metabolic pathways for improving chemotherapy and immunotherapy. Cancer Lett. (2023) 575:216396. doi: 10.1016/j.canlet.2023.216396, PMID: 37739209 PMC10591810

[12] Chen DQ Xie Y Cao LQ Fleishman JS Chen Y Wu T . The role of abcc10/mrp7 in anti-cancer drug resistance and beyond. Drug Resist Updat. (2024) 73:101062. doi: 10.1016/j.drup.2024.101062, PMID: 38330827

[13] Liu J Fan H Liang X Chen Y . Polycomb repressor complex: Its function in human cancer and therapeutic target strategy. BioMed Pharmacother. (2023) 169:115897. doi: 10.1016/j.biopha.2023.115897, PMID: 37981459

[14] Gao L Zhang J Long Q Yang Y Li Y Li G . Setd7 promotes metastasis of triple-negative breast cancer by yy1 lysine methylation. Biochim Biophys Acta Mol Basis Dis. (2023) 1869:166780. doi: 10.1016/j.bbadis.2023.166780, PMID: 37286143

[15] Faubert B Solmonson A DeBerardinis RJ . Metabolic reprogramming and cancer progression. Science. (2020) 368:eaaw5473. doi: 10.1126/science.aaw5473, PMID: 32273439 PMC7227780

[16] Warburg O . On respiratory impairment in cancer cells. Science. (1956) 124:269–270. Available online at: https://pubmed.ncbi.nlm.nih.gov/13351639/. 13351639

[17] Zhu Q Li J Sun H Fan Z Hu J Chai S . O-glcnacylation of enolase 1 serves as a dual regulator of aerobic glycolysis and immune evasion in colorectal cancer. Proc Natl Acad Sci U S A. (2024) 121:e2408354121. doi: 10.1073/pnas.2408354121, PMID: 39446384 PMC11536113

[18] Du D Liu C Qin M Zhang X Xi T Yuan S . Metabolic dysregulation and emerging therapeutical targets for hepatocellular carcinoma. Acta Pharm Sin B. (2022) 12:558–580. doi: 10.1016/j.apsb.2021.09.019, PMID: 35256934 PMC8897153

[19] Zhong J Tian L Gou Y Zhao P Dong X Guo M . BMP4 upregulates glycogen synthesis through the SMAD/SLC2A1 (GLUT1) signaling axis in hepatocellular carcinoma (HCC) cells. Cancer Metab. (2023) 11:9. doi: 10.1186/s40170-023-00310-6, PMID: 37443106 PMC10339511

[20] De Giovanni M Tam H Valet C Xu Y Looney MR Cyster JG . GPR35 promotes neutrophil recruitment in response to serotonin metabolite 5-HIAA. CELL. (2022) 185:815–830.e819. doi: 10.1016/j.cell.2022.01.010, PMID: 35148838 PMC9037118

[21] Agudelo LZ Ferreira DMS Cervenka I Bryzgalova G Dadvar S Jannig PR . Kynurenic acid and Gpr35 regulate adipose tissue energy homeostasis and inflammation. Cell Metab. (2018) 27:378–392.e375. doi: 10.1016/j.cmet.2018.01.004, PMID: 29414686

[22] Wu Y Zhang P Fan H Zhang C Yu P Liang X . GPR35 acts a dual role and therapeutic target in inflammation. Front Immunol. (2023) 14:1254446. doi: 10.3389/fimmu.2023.1254446, PMID: 38035084 PMC10687457

[23] Otkur W Zhang Y Li Y Bao W Feng T Wu B . Spatial multi-omics characterizes GPR35-relevant lipid metabolism signatures across liver zonation in MASLD. Life Metab. (2024) 3:loae021. doi: 10.1093/lifemeta/loae021, PMID: 39873004 PMC11748505

[24] Zhang H Fan H Wang J Hou T Saravanan KM Xia W . Revolutionizing GPCR-ligand predictions: Deepgpcr with experimental validation for high-precision drug discovery. Brief Bioinform. (2024) 25:bbae281. doi: 10.1093/bib/bbae281, PMID: 38864340 PMC11167311

[25] Nam SY Park SJ Im DS . Protective effect of lodoxamide on hepatic steatosis through GPR35. Cell Signal. (2019) 53:190–200. doi: 10.1016/j.cellsig.2018.10.001, PMID: 30304698

[26] Schneditz G Elias JE Pagano E Zaeem Cader M Saveljeva S Long K . GPR35 promotes glycolysis, proliferation, and oncogenic signaling by engaging with the sodium potassium pump. Sci Signal. (2019) 12:eaau9048. doi: 10.1126/scisignal.aau9048, PMID: 30600262 PMC6364804

[27] Wang D Li D Zhang Y Chen J Zhang Y Liao C . Functional metabolomics reveal the role of AHR/GPR35 mediated kynurenic acid gradient sensing in chemotherapy-induced intestinal damage. Acta Pharm Sin B. (2021) 11:763–780. doi: 10.1016/j.apsb.2020.07.017, PMID: 33777681 PMC7982426

[28] Takkar S Sharma G Kaushal JB Abdullah KM Batra SK Siddiqui JA . From orphan to oncogene: The role of GPR35 in cancer and immune modulation. Cytokine Growth Factor Rev. (2024) 77:56–66. doi: 10.1016/j.cytogfr.2024.03.004, PMID: 38514303 PMC11793123

[29] Milligan G . GPR35: From enigma to therapeutic target. Trends Pharmacol Sci. (2023) 44:263–273. doi: 10.1016/j.tips.2023.03.001, PMID: 37002007

[30] Pagano E Elias JE Schneditz G Saveljeva S Holland LM Borrelli F . Activation of the GPR35 pathway drives angiogenesis in the tumour microenvironment. GUT. (2022) 71:509–520. doi: 10.1136/gutjnl-2020-323363, PMID: 33758004 PMC8862021

[31] Shu C Wang C Chen S Huang X Cui J Li W . ERR-activated GPR35 promotes immune infiltration level of macrophages in gastric cancer tissues. Cell Death Discov. (2022) 8:444. doi: 10.1038/s41420-022-01238-4, PMID: 36333291 PMC9636254

[32] Zhang QQ Zhao X Qin SY Xiao QW Tian Y Zhang ZJ . Identification of GPR35-associated metabolic characteristics through lc-ms/ms-based metabolomics and lipidomics. Acta Materia Medica. (2024) 3:105–118. doi: 10.15212/AMM-2023-0046

[33] Duan J Liu Q Yuan Q Ji Y Zhu S Tan Y . Insights into divalent cation regulation and G_13_-coupling of orphan receptor GPR35. Cell Discov. (2022) 8:135. doi: 10.1038/s41421-022-00499-8, PMID: 36543774 PMC9772185

[34] Shore DM Reggio PH . The therapeutic potential of orphan GPCRs, GPR35 and GPR55. Front Pharmacol. (2015) 6:69. doi: 10.3389/fphar.2015.00069, PMID: 25926795 PMC4397721

[35] Quon T Lin LC Ganguly A Tobin AB Milligan G . Therapeutic opportunities and challenges in targeting the orphan G Protein-coupled receptor GPR35. ACS Pharmacol Transl Sci. (2020) 3:801–812. doi: 10.1021/acsptsci.0c00079, PMID: 33073184 PMC7551713

[36] Divorty N Jenkins L Ganguly A Butcher AJ Hudson BD Schulz S . Agonist-induced phosphorylation of orthologues of the orphan receptor GPR35 functions as an activation sensor. J Biol Chem. (2022) 298:101655. doi: 10.1016/j.jbc.2022.101655, PMID: 35101446 PMC8892012

[37] Wei L Xiang K Kang H Yu Y Fan H Zhou H . Development and characterization of fluorescent probes for the G Protein-coupled receptor 35. ACS Med Chem Lett. (2023) 14:411–416. doi: 10.1021/acsmedchemlett.2c00461, PMID: 37077394 PMC10107913

[38] Fallarini S Magliulo L Paoletti T de Lalla C Lombardi G . Expression of functional GPR35 in human iNKT cells. Biochem Biophys Res Commun. (2010) 398:420–425. doi: 10.1016/j.bbrc.2010.06.091, PMID: 20599711

[39] Guo J Williams DJ Puhl HL 3rd Ikeda SR . Inhibition of N-type calcium channels by activation of GPR35, an orphan receptor, heterologously expressed in rat sympathetic neurons. J Pharmacol Exp Ther. (2008) 324:342–351. doi: 10.1124/jpet.107.127266, PMID: 17940199

[40] Zhao P Abood ME . GPR55 and GPR35 and their relationship to cannabinoid and lysophospholipid receptors. Life Sci. (2013) 92:453–457. doi: 10.1016/j.lfs.2012.06.039, PMID: 22820167

[41] Wang J Simonavicius N Wu X Swaminath G Reagan J Tian H . Kynurenic acid as a ligand for orphan G protein-coupled receptor GPR35. J Biol Chem. (2006) 281:22021–22028. doi: 10.1074/jbc.M603503200, PMID: 16754668

[42] Maravillas-Montero JL Burkhardt AM Hevezi PA Carnevale CD Smit MJ Zlotnik A . Cutting edge: GPR35/CXCR8 is the receptor of the mucosal chemokine CXCL17. J Immunol. (2015) 194:29–33. doi: 10.4049/jimmunol.1401704, PMID: 25411203 PMC4355404

[43] Zhang S Sun Z Chen Z Bi Y Wei S Mao Z . Endothelial yap/tead1-CXCL17 signaling recruits myeloid-derived suppressor cells against liver ischemia-reperfusion injury. Hepatology. (2025) 81:888–902. doi: 10.1097/hep.0000000000000773, PMID: 38407233 PMC11825485

[44] Bu J Yan W Huang Y Lin K . Activation of the IL-17 signalling pathway by the CXCL17-GPR35 axis affects drug resistance and colorectal cancer tumorigenesis. Am J Cancer Res. (2023) 13:2172–2187. https://pubmed.ncbi.nlm.nih.gov/37293165/., PMID: 37293165 PMC10244108

[45] Cosi C Mannaioni G Cozzi A Carlà V Sili M Cavone L . G-protein coupled receptor 35 (GPR35) activation and inflammatory pain: Studies on the antinociceptive effects of kynurenic acid and zaprinast. Neuropharmacology. (2011) 60:1227–1231. doi: 10.1016/j.neuropharm.2010.11.014, PMID: 21110987

[46] Im DS . Recent advances in GPR35 pharmacology; 5-HIAA serotonin metabolite becomes a ligand. Arch Pharm Res. (2023) 46:550–563. doi: 10.1007/s12272-023-01449-y, PMID: 37227682

[47] Foata F Sprenger N Rochat F Damak S . Activation of the G-protein coupled receptor GPR35 by human milk oligosaccharides through different pathways. Sci Rep. (2020) 10:16117. doi: 10.1038/s41598-020-73008-0, PMID: 32999316 PMC7528069

[48] Zhang C Zhang H Zhang Q Fan H Yu P Xia W . Targeting atp catalytic activity of chromodomain helicase chd1l for the anticancer inhibitor discovery. Int J Biol Macromol. (2024) 281:136678. doi: 10.1016/j.ijbiomac.2024.136678, PMID: 39426766

[49] Xia C Dong X Li H Cao M Sun D He S . Cancer statistics in China and United States, 2022: Profiles, trends, and determinants. Chin Med J (Engl). (2022) 135:584–590. doi: 10.1097/cm9.0000000000002108, PMID: 35143424 PMC8920425

[50] Li L Yan J Xu J Liu CQ Zhen ZJ Chen HW . CXCL17 expression predicts poor prognosis and correlates with adverse immune infiltration in hepatocellular carcinoma. PloS One. (2014) 9:e110064. doi: 10.1371/journal.pone.0110064, PMID: 25303284 PMC4193880

[51] Wang L Li H Zhen Z Ma X Yu W Zeng H . CXCL17 promotes cell metastasis and inhibits autophagy via the LKB1-AMPK pathway in hepatocellular carcinoma. Gene. (2019) 690:129–136. doi: 10.1016/j.gene.2018.12.043, PMID: 30597237

[52] Li Y Liu A Liu S Yan L Yuan Y Xu Q . Involvement of CXCL17 and GPR35 in gastric cancer initiation and progression. Int J Mol Sci. (2022) 24:615. doi: 10.3390/ijms24010615, PMID: 36614059 PMC9820077

[53] Okumura S Baba H Kumada T Nanmoku K Nakajima H Nakane Y . Cloning of a G-protein-coupled receptor that shows an activity to transform NIH3T3 cells and is expressed in gastric cancer cells. Cancer Sci. (2004) 95:131–135. doi: 10.1111/j.1349-7006.2004.tb03193.x, PMID: 14965362 PMC11159784

[54] Mackiewicz T Włodarczyk J Zielińska M Włodarczyk M Durczyński A Hogendorf P . Increased GPR35 expression in human colorectal and pancreatic cancer samples: A preliminary clinical validation of a new biomarker. Adv Clin Exp Med. (2023) 32:783–789. doi: 10.17219/acem/157291, PMID: 36637186

[55] Xiang Q Zhou D Xiang X Le X Deng C Sun R . Neuroglobin plays as tumor suppressor by disrupting the stability of GPR35 in colorectal cancer. Clin Epigenetics. (2023) 15:57. doi: 10.1186/s13148-023-01472-2, PMID: 37005662 PMC10067258

[56] Otkur W Liu X Chen H Li S Ling T Lin H . GPR35 antagonist CID-2745687 attenuates anchorage-independent cell growth by inhibiting YAP/TAZ activity in colorectal cancer cells. Front Pharmacol. (2023) 14:1126119. doi: 10.3389/fphar.2023.1126119, PMID: 37113762 PMC10126512

[57] Alahdal M Sun D Duan L Ouyang H Wang M Xiong J . Forecasting sensitive targets of the kynurenine pathway in pancreatic adenocarcinoma using mathematical modeling. Cancer Sci. (2021) 112:1481–1494. doi: 10.1111/cas.14832, PMID: 33523522 PMC8019197

[58] Kim M . The role of protein-coupled receptor 35 in pancreatic cancer . Perth, Australia Curtin University. Available online at: http://hdl.handle.net/20.500.11937/84527 (Accessed June 3, 2022).

[59] Hiraoka N Yamazaki-Itoh R Ino Y Mizuguchi Y Yamada T Hirohashi S . CXCL17 and ICAM2 are associated with a potential anti-tumor immune response in early intraepithelial stages of human pancreatic carcinogenesis. Gastroenterology. (2011) 140:310–321. doi: 10.1053/j.gastro.2010.10.009, PMID: 20955708

[60] Thrift AP Wenker TN El-Serag HB . Global burden of gastric cancer: Epidemiological trends, risk factors, screening and prevention. Nat Rev Clin Oncol. (2023) 20:338–349. doi: 10.1038/s41571-023-00747-0, PMID: 36959359

[61] Hindson J . Nivolumab plus chemotherapy for advanced gastric cancer and oesophageal adenocarcinoma. Nat Rev Gastroenterol Hepatol. (2021) 18:523. doi: 10.1038/s41575-021-00484-8, PMID: 34158606

[62] Hu J Cao J Huang S Chen Y . Itgax promotes gastric cancer progression via epithelial-mesenchymal transition pathway. Front Pharmacol. (2025) 15:1536478. doi: 10.3389/fphar.2024.1536478, PMID: 39845786 PMC11750855

[63] Liu Y Guo S Yuan T Chen Y . Editorial: Novel advances in gastrointestinal cancer treatment. Front Mol Biosci. (2023) 10:1238098. doi: 10.3389/fmolb.2023.1238098, PMID: 37457830 PMC10348898

[64] Jia SN Han YB Yang R Yang ZC . Chemokines in colon cancer progression. Semin Cancer Biol. (2022) 86:400–407. doi: 10.1016/j.semcancer.2022.02.007, PMID: 35183412

[65] Chen W Qin Y Liu S . CCL20 signaling in the tumor microenvironment. Adv Exp Med Biol. (2020) 1231:53–65. doi: 10.1007/978-3-030-36667-4_6, PMID: 32060846

[66] Wang YN Lu YX Liu J Jin Y Bi HC Zhao Q . Ampkα1 confers survival advantage of colorectal cancer cells under metabolic stress by promoting redox balance through the regulation of glutathione reductase phosphorylation. Oncogene. (2020) 39:637–650. doi: 10.1038/s41388-019-1004-2, PMID: 31530934 PMC6962094

[67] Luo S Yue M Wang D Lu Y Wu Q Jiang J . Breaking the barrier: Epigenetic strategies to combat platinum resistance in colorectal cancer. Drug Resist Updat. (2024) 77:101152. doi: 10.1016/j.drup.2024.101152, PMID: 39369466

[68] Wang Z Chen Y Huang A Wen H Wu Y Xu X . Design, synthesis and biological evaluation of novel beta-caryophyllene derivatives as potential anti-cancer agents through the ros-mediated apoptosis pathway. RSC Med Chem. (2025). doi: 10.1039/d4md00951g, PMID: 40352675 PMC12061030

[69] Ali H AbdelMageed M Olsson L Israelsson A Lindmark G Hammarström ML . Utility of G protein-coupled receptor 35 expression for predicting outcome in colon cancer. Tumour Biol. (2019) 41:1010428319858885. doi: 10.1177/1010428319858885, PMID: 31250711

[70] Mackiewicz T Jacenik D Talar M Fichna J . The GPR35 expression pattern is associated with overall survival in male patients with colorectal cancer. Pharmacol Rep. (2022) 74:709–717. doi: 10.1007/s43440-022-00371-2, PMID: 35622222

[71] Yao H Lv Y Bai X Yu Z Liu X . Prognostic value of CXCL17 and CXCR8 expression in patients with colon cancer. Oncol Lett. (2020) 20:2711–2720. doi: 10.3892/ol.2020.11819, PMID: 32782587 PMC7400977

[72] Yu B Shao S Ma W . Frontiers in pancreatic cancer on biomarkers, microenvironment, and immunotherapy. Cancer Lett. (2025) 610:217350. doi: 10.1016/j.canlet.2024.217350, PMID: 39581219

[73] Shah A Ganguly K Rauth S Sheree SS Khan I Ganti AK . Unveiling the resistance to therapies in pancreatic ductal adenocarcinoma. Drug Resist Updat. (2024) 77:101146. doi: 10.1016/j.drup.2024.101146, PMID: 39243602 PMC11770815

[74] Fan H Zhao H Gao L Dong Y Zhang P Yu P . Ccn1 enhances tumor immunosuppression through collagen-mediated chemokine secretion in pancreatic cancer. Advanced Sci (Weinheim Baden-Wurttemberg Germany). (2025) 12:e2500589. doi: 10.1002/advs.202500589, PMID: 40287974 PMC12199403

[75] Guo S Xie X Chen Y Liu Y Luo L . Editorial: Advances of novel approaches to enhance therapeutic efficacy and safety in human solid cold tumor. Front Immunol. (2024) 15:1398270. doi: 10.3389/fimmu.2024.1398270, PMID: 38585262 PMC10995367

[76] Botwinick IC Pursell L Yu G Cooper T Mann JJ Chabot JA . A biological basis for depression in pancreatic cancer. HPB (Oxford). (2014) 16:740–743. doi: 10.1111/hpb.12201, PMID: 24467653 PMC4113256

[77] Wei X Yin F Wu M Xie Q Zhao X Zhu C . G protein-coupled receptor 35 attenuates nonalcoholic steatohepatitis by reprogramming cholesterol homeostasis in hepatocytes. Acta Pharm Sin B. (2023) 13:1128–1144. doi: 10.1016/j.apsb.2022.10.011, PMID: 36970193 PMC10031266

[78] Bao L Zhang Y Yan S Yan D Jiang D . Lysophosphatidylcholine (17:0) improves HFD-induced hyperglycemia & insulin resistance: A mechanistic mice model study. Diabetes Metab Syndr Obes. (2022) 15:3511–3517. doi: 10.2147/dmso.S371370, PMID: 36411788 PMC9675350

[79] Wu X Chen S Yan Q Yu F Shao H Zheng X . Gpr35 shapes gut microbial ecology to modulate hepatic steatosis. Pharmacol Res. (2023) 189:106690. doi: 10.1016/j.phrs.2023.106690, PMID: 36758734

[80] Clemente-Suárez VJ Mielgo-Ayuso J Martín-Rodríguez A Ramos-Campo DJ Redondo-Flórez L Tornero-Aguilera JF . The burden of carbohydrates in health and disease. Nutrients. (2022) 14:3809. doi: 10.3390/nu14183809, PMID: 36145184 PMC9505863

[81] Lin LC Quon T Engberg S Mackenzie AE Tobin AB Milligan G . G Protein-coupled receptor GPR35 suppresses lipid accumulation in hepatocytes. ACS Pharmacol Transl Sci. (2021) 4:1835–1848. doi: 10.1021/acsptsci.1c00224, PMID: 34927014 PMC8669712

[82] Kałużna-Czaplińska J Gątarek P Chirumbolo S Chartrand MS Bjørklund G . How important is tryptophan in human health? Crit Rev Food Sci Nutr. (2019) 59:72–88. doi: 10.1080/10408398.2017.1357534, PMID: 28799778

[83] Vécsei L Szalárdy L Fülöp F Toldi J . Kynurenines in the CNS: Recent advances and new questions. Nat Rev Drug Discov. (2013) 12:64–82. doi: 10.1038/nrd3793, PMID: 23237916

[84] Cervenka I Agudelo LZ Ruas JL . Kynurenines: Tryptophan’s metabolites in exercise, inflammation, and mental health. Science. (2017) 357:eaaf9794. doi: 10.1126/science.aaf9794, PMID: 28751584

[85] Wang Y Liu Z Shen P Zhao C Liu B Shu C . Kynurenic acid ameliorates lipopolysaccharide-induced endometritis by regulating the GRP35/NF-κB signaling pathway. Toxicol Appl Pharmacol. (2022) 438:115907. doi: 10.1016/j.taap.2022.115907, PMID: 35123988

[86] Barth MC Ahluwalia N Anderson TJ Hardy GJ Sinha S Alvarez-Cardona JA . Kynurenic acid triggers firm arrest of leukocytes to vascular endothelium under flow conditions. J Biol Chem. (2009) 284:19189–19195. doi: 10.1074/jbc.M109.024042, PMID: 19473985 PMC2740542

[87] Wyant GA Yu W Doulamis IP Nomoto RS Saeed MY Duignan T . Mitochondrial remodeling and ischemic protection by G protein-coupled receptor 35 agonists. Science. (2022) 377:621–629. doi: 10.1126/science.abm1638, PMID: 35926043 PMC9639781

[88] Zhang X Cai X Zheng X . Gut microbiome-oriented therapy for metabolic diseases: Challenges and opportunities towards clinical translation. Trends Pharmacol Sci. (2021) 42:984–987. doi: 10.1016/j.tips.2021.09.003, PMID: 34579969

[89] Hou Y Wei W Guan X Liu Y Bian G He D . A diet-microbial metabolism feedforward loop modulates intestinal stem cell renewal in the stressed gut. Nat Commun. (2021) 12:271. doi: 10.1038/s41467-020-20673-4, PMID: 33431867 PMC7801547

[90] Li Y Zhao D Qian M Liu J Pan C Zhang X . Amlodipine, an anti-hypertensive drug, alleviates non-alcoholic fatty liver disease by modulating gut microbiota. Br J Pharmacol. (2022) 179:2054–2077. doi: 10.1111/bph.15768, PMID: 34862599

[91] Fan Y Pedersen O . Gut microbiota in human metabolic health and disease. Nat Rev Microbiol. (2021) 19:55–71. doi: 10.1038/s41579-020-0433-9, PMID: 32887946

[92] Lund ML Egerod KL Engelstoft MS Dmytriyeva O Theodorsson E Patel BA . Enterochromaffin 5-HT cells - a major target for GLP-1 and gut microbial metabolites. Mol Metab. (2018) 11:70–83. doi: 10.1016/j.molmet.2018.03.004, PMID: 29576437 PMC6001397

[93] Cheng L Wu H Cai X Zhang Y Yu S Hou Y . A Gpr35-tuned gut microbe-brain metabolic axis regulates depressive-like behavior. Cell Host Microbe. (2024) 32:227–243.e226. doi: 10.1016/j.chom.2023.12.009, PMID: 38198925

[94] Wang D Wang W Bing X Xu C Qiu J Shen J . GPR35-mediated kynurenic acid sensing contributes to maintenance of gut microbiota homeostasis in ulcerative colitis. FEBS Open Bio. (2023) 13:1415–1433. doi: 10.1002/2211-5463.13673, PMID: 37423235 PMC10392069

[95] Boleij A Fathi P Dalton W Park B Wu X Huso D . G-protein coupled receptor 35 (GPR35) regulates the colonic epithelial cell response to enterotoxigenic bacteroides fragilis. Commun Biol. (2021) 4:585. doi: 10.1038/s42003-021-02014-3, PMID: 33990686 PMC8121840

[96] Kaya B Doñas C Wuggenig P Diaz OE Morales RA Melhem H . Lysophosphatidic acid-mediated GPR35 signaling in CX3CR1^+^ macrophages regulates intestinal homeostasis. Cell Rep. (2020) 32:107979. doi: 10.1016/j.celrep.2020.107979, PMID: 32755573

[97] Jia W Yu S Liu X Le Q He X Yu L . Ethanol extract of *limonium bicolor* improves dextran sulfate sodium-induced ulcerative colitis by alleviating inflammation and restoring gut microbiota dysbiosis in mice. Mar Drugs. (2024) 22:175. doi: 10.3390/md22040175, PMID: 38667792 PMC11050939

[98] Lee JY Hwang HW Jin HS Lee JE Kang NJ Lee DW . A Genomics-Based Semirational approach for expanding the postbiotic potential of collagen peptides using Lactobacillaceae. J Agric Food Chem. (2022) 70:8365–8376. doi: 10.1021/acs.jafc.2c01251, PMID: 35758868

[99] Miyamoto K Sujino T Harada Y Ashida H Yoshimatsu Y Yonemoto Y . The gut microbiota-induced kynurenic acid recruits GPR35-positive macrophages to promote experimental encephalitis. Cell Rep. (2023) 42:113005. doi: 10.1016/j.celrep.2023.113005, PMID: 37590143

[100] Pavlova NN Thompson CB . The emerging hallmarks of cancer metabolism. Cell Metab. (2016) 23:27–47. doi: 10.1016/j.cmet.2015.12.006, PMID: 26771115 PMC4715268

[101] Marcucci F Rumio C . On the role of glycolysis in early tumorigenesis-permissive and executioner effects. Cells. (2023) 12:1124. doi: 10.3390/cells12081124, PMID: 37190033 PMC10137279

[102] Vaupel P Schmidberger H Mayer A . The Warburg effect: Essential part of metabolic reprogramming and central contributor to cancer progression. Int J Radiat Biol. (2019) 95:912–919. doi: 10.1080/09553002.2019.1589653, PMID: 30822194

[103] Kooshan Z Cárdenas-Piedra L Clements J Batra J . Glycolysis, the sweet appetite of the tumor microenvironment. Cancer Lett. (2024) 600:217156. doi: 10.1016/j.canlet.2024.217156, PMID: 39127341

[104] Cai L Pessoa MT Gao Y Strause S Banerjee M Tian J . The Na/K-ATPase α1/Src signaling axis regulates mitochondrial metabolic function and redox signaling in human iPSC-derived cardiomyocytes. Biomedicines. (2023) 11:3207. doi: 10.3390/biomedicines11123207, PMID: 38137428 PMC10740578

[105] Cong J Liu P Han Z Ying W Li C Yang Y . Bile acids modified by the intestinal microbiota promote colorectal cancer growth by suppressing CD8^+^ t cell effector functions. Immunity. (2024) 57:876–889.e811. doi: 10.1016/j.immuni.2024.02.014, PMID: 38479384

[106] Ala M . The footprint of kynurenine pathway in every cancer: A new target for chemotherapy. Eur J Pharmacol. (2021) 896:173921. doi: 10.1016/j.ejphar.2021.173921, PMID: 33529725

[107] Chung L Orberg ET Geis AL Chan JL Fu K DeStefano Shields CE . Bacteroides fragilis toxin coordinates a pro-carcinogenic inflammatory cascade via targeting of colonic epithelial cells. Cell Host Microbe. (2018) 23:421. doi: 10.1016/j.chom.2018.02.004, PMID: 29544099 PMC6469393

[108] Song Y Lee SY Kim S Choi I Kim SH Shum D . Inhibitors of Na^+^/K^+^ ATPase exhibit antitumor effects on multicellular tumor spheroids of hepatocellular carcinoma. Sci Rep. (2020) 10:5318. doi: 10.1038/s41598-020-62134-4, PMID: 32210281 PMC7093469

[109] Hua S Wang X Chen F Gou S . Novel conjugates with dual suppression of glutathione s-transferases and tryptophan-2,3-dioxygenase activities for improving hepatocellular carcinoma therapy. Bioorg Chem. (2019) 92:103191. doi: 10.1016/j.bioorg.2019.103191, PMID: 31445192

[110] Nakamura K Shiozaki A Kosuga T Shimizu H Kudou M Ohashi T . The expression of the alpha1 subunit of Na^+^/K^+^-ATPase is related to tumor development and clinical outcomes in gastric cancer. Gastric Cancer. (2021) 24:1278–1292. doi: 10.1007/s10120-021-01212-6, PMID: 34251542

[111] Wang S Kuang J Zhang H Chen W Zheng X Wang J . Bile acid-microbiome interaction promotes gastric carcinogenesis. Adv Sci (Weinh). (2022) 9:e2200263. doi: 10.1002/advs.202200263, PMID: 35285172 PMC9165488

[112] Li F Sun Y Huang J Xu W Liu J Yuan Z . CD4/cd8 + t cells, DC subsets, Foxp3, and IDO expression are predictive indictors of gastric cancer prognosis. Cancer Med. (2019) 8:7330–7344. doi: 10.1002/cam4.2596, PMID: 31631566 PMC6885892

[113] Wu J Zhang R Yin Z Chen X Mao R Zheng X . Gut microbiota-driven metabolic alterations reveal the distinct pathogenicity of chemotherapy-induced cachexia in gastric cancer. Pharmacol Res. (2024) 209:107476. doi: 10.1016/j.phrs.2024.107476, PMID: 39490563

[114] Xu Z Huang Z Zhang Y Sun H Hinz U Heger U . Farnesoid X receptor activation inhibits pancreatic carcinogenesis. Biochim Biophys Acta Mol Basis Dis. (2023) 1869:166811. doi: 10.1016/j.bbadis.2023.166811, PMID: 37515840 PMC10935600

[115] Chen L Jiang P Li J Xie Z Xu Y Qu W . Periplocin promotes wound healing through the activation of Src/ERK and PI3K/Akt pathways mediated by Na/K-ATPase. Phytomedicine. (2019) 57:72–83. doi: 10.1016/j.phymed.2018.12.015, PMID: 30668325

[116] Khajah MA Mathew PM Luqmani YA . Na^+^/K^+^ ATPase activity promotes invasion of endocrine resistant breast cancer cells. PloS One. (2018) 13:e0193779. doi: 10.1371/journal.pone.0193779, PMID: 29590154 PMC5874017

[117] Ren Y Anderson AT Meyer G Lauber KM Gallucci JC Douglas Kinghorn A . Digoxin and its Na^+^/K^+^-ATPase-targeted actions on cardiovascular diseases and cancer. Bioorg Med Chem. (2024) 114:117939. doi: 10.1016/j.bmc.2024.117939, PMID: 39396465 PMC11527570

[118] Ren Y Wu S Burdette JE Cheng X Kinghorn AD . Structural insights into the interactions of digoxin and Na^+^/K^+^-ATPase and other targets for the inhibition of cancer cell proliferation. Molecules. (2021) 26:3672. doi: 10.3390/molecules26123672, PMID: 34208576 PMC8234910

[119] Harich OO Gavriliuc OI Ordodi VL Tirziu A Paunescu V Panaitescu C . *In vitro* study of the multimodal effect of Na^+^/K^+^ ATPase blocker ouabain on the tumor microenvironment and Malignant cells. Biomedicines. (2023) 11:2205. doi: 10.3390/biomedicines11082205, PMID: 37626702 PMC10452365

[120] Jin HR Wang J Wang ZJ Xi MJ Xia BH Deng K . Lipid metabolic reprogramming in tumor microenvironment: From mechanisms to therapeutics. J Hematol Oncol. (2023) 16:103. doi: 10.1186/s13045-023-01498-2, PMID: 37700339 PMC10498649

[121] Wang W Bai L Li W Cui J . The lipid metabolic landscape of cancers and new therapeutic perspectives. Front Oncol. (2020) 10:605154. doi: 10.3389/fonc.2020.605154, PMID: 33364199 PMC7753360

[122] Fuchs CD Trauner M . Role of bile acids and their receptors in gastrointestinal and hepatic pathophysiology. Nat Rev Gastroenterol Hepatol. (2022) 19:432–450. doi: 10.1038/s41575-021-00566-7, PMID: 35165436

[123] Conde de la Rosa L Garcia-Ruiz C Vallejo C Baulies A Nuñez S Monte MJ . STARD1 promotes NASH-driven HCC by sustaining the generation of bile acids through the alternative mitochondrial pathway. J Hepatol. (2021) 74:1429–1441. doi: 10.1016/j.jhep.2021.01.028, PMID: 33515644 PMC8573791

[124] Wahlström A Sayin SI Marschall HU Bäckhed F . Intestinal crosstalk between bile acids and microbiota and its impact on host metabolism. Cell Metab. (2016) 24:41–50. doi: 10.1016/j.cmet.2016.05.005, PMID: 27320064

[125] Girisa S Henamayee S Parama D Rana V Dutta U Kunnumakkara AB . Targeting Farnesoid X receptor (FXR) for developing novel therapeutics against cancer. Mol Biomed. (2021) 2:21. doi: 10.1186/s43556-021-00035-2, PMID: 35006466 PMC8607382

[126] Lemos H Huang L Prendergast GC Mellor AL . Immune control by amino acid catabolism during tumorigenesis and therapy. Nat Rev Cancer. (2019) 19:162–175. doi: 10.1038/s41568-019-0106-z, PMID: 30696923

[127] De Giovanni M Dang EV Chen KY An J Madhani HD Cyster JG . Platelets and mast cells promote pathogenic eosinophil recruitment during invasive fungal infection via the 5-HIAA-GPR35 ligand-receptor system. IMMUNITY. (2023) 56:1548–1560 e1545. doi: 10.1016/j.immuni.2023.05.006, PMID: 37279752 PMC10360074

[128] Xiong S Dong L Cheng L . Neutrophils in cancer carcinogenesis and metastasis. J Hematol Oncol. (2021) 14:173. doi: 10.1186/s13045-021-01187-y, PMID: 34674757 PMC8529570

[129] Fan H Zhao H Zheng Y Chen G Ji Y Yu W . Polygonati kingianum polysaccharide alleviates dextran sulfate sodium-induced colitis by modulating gut microbiota and metabolic homeostasis. Int J Biol Macromol. (2025) 316:144836. doi: 10.1016/j.ijbiomac.2025.144836, PMID: 40451347

[130] Li P Lu M Shi J Gong Z Hua L Li Q . Lung mesenchymal cells elicit lipid storage in neutrophils that fuel breast cancer lung metastasis. Nat Immunol. (2020) 21:1444–1455. doi: 10.1038/s41590-020-0783-5, PMID: 32958928 PMC7584447

[131] Pan L Li R Li Q Zhu Q Zhou Q Su A . The gut-brain axis mechanism of normal appetite induced by kynurenic acid. Cell Rep. (2025) 44:115659. doi: 10.1016/j.celrep.2025.115659, PMID: 40317720

[132] Poźniak O Sitarz R Sitarz MZ Kowalczuk D Słoń E Dudzińska E . Utilization of ahr and GPR35 receptor ligands as superfoods in cancer prevention for individuals with ibd. Int J Mol Sci. (2025) 26:9160. doi: 10.3390/ijms26189160, PMID: 41009721 PMC12470665

[133] Badawy AA . Tryptophan metabolism and disposition in cancer biology and immunotherapy. Biosci Rep. (2022) 42:BSR20221682. doi: 10.1042/bsr20221682, PMID: 36286592 PMC9653095

[134] Cavia-Saiz M Muñiz P De Santiago R Herreros-Villanueva M Garcia-Giron C Lopez AS . Changes in the levels of thioredoxin and indoleamine-2,3-dioxygenase activity in plasma of patients with colorectal cancer treated with chemotherapy. Biochem Cell Biol. (2012) 90:173–178. doi: 10.1139/o11-077, PMID: 22257103

[135] Engin AB Karahalil B Karakaya AE Engin A . Helicobacter pylori and serum kynurenine-tryptophan ratio in patients with colorectal cancer. World J Gastroenterol. (2015) 21:3636–3643. doi: 10.3748/wjg.v21.i12.3636, PMID: 25834331 PMC4375588

[136] He H Kim NH Han S Chu ZL . Abstract lb426: Evidence for a role of GPR35 in ido1-mediated tumor immune escape by regulating hippo-yap pathway. Cancer Res. (2024) 84. doi: 10.1158/1538-7445.AM2024-LB426

[137] Bekki S Hashimoto S Yamasaki K Komori A Abiru S Nagaoka S . Serum kynurenine levels are a novel biomarker to predict the prognosis of patients with hepatocellular carcinoma. PloS One. (2020) 15:e0241002. doi: 10.1371/journal.pone.0241002, PMID: 33085694 PMC7577466

[138] Li S Li L Wu J Song F Qin Z Hou L . TDO promotes hepatocellular carcinoma progression. Onco Targets Ther. (2020) 13:5845–5855. doi: 10.2147/ott.S252929, PMID: 32606795 PMC7311207

[139] Peng YP Zhang JJ Liang WB Tu M Lu ZP Wei JS . Elevation of MMP-9 and IDO induced by pancreatic cancer cells mediates natural killer cell dysfunction. BMC Cancer. (2014) 14:738. doi: 10.1186/1471-2407-14-738, PMID: 25274283 PMC4287420

[140] Elias JE Debela M Sewell GW Stopforth RJ Partl H Heissbauer S . GPR35 prevents osmotic stress induced cell damage. Commun Biol. (2025) 8:478. doi: 10.1038/s42003-025-07848-9, PMID: 40121360 PMC11929815

[141] Sobhani I Bergsten E Couffin S Amiot A Nebbad B Barau C . Colorectal cancer-associated microbiota contributes to oncogenic epigenetic signatures. Proc Natl Acad Sci U S A. (2019) 116:24285–24295. doi: 10.1073/pnas.1912129116, PMID: 31712445 PMC6883805

[142] Qu R Zhang Y Kim B Zeng G Wang P Shaoyong W . Microbial riboflavin inhibits ceramide synthase 3 to lower ceramide (d18:1/26:0) and delay colorectal cancer progression. Cell Metab. (2025) 37:1852–1869.e1858. doi: 10.1016/j.cmet.2025.06.002, PMID: 40609532 PMC12365777

[143] Arthur JC Perez-Chanona E Mühlbauer M Tomkovich S Uronis JM Fan TJ . Intestinal inflammation targets cancer-inducing activity of the microbiota. Science. (2012) 338:120–123. doi: 10.1126/science.1224820, PMID: 22903521 PMC3645302

[144] Drewes JL White JR Dejea CM Fathi P Iyadorai T Vadivelu J . High-resolution bacterial 16s rRNA gene profile meta-analysis and biofilm status reveal common colorectal cancer consortia. NPJ Biofilms Microbiomes. (2017) 3:34. doi: 10.1038/s41522-017-0040-3, PMID: 29214046 PMC5707393

[145] Kim MJ Park SJ Nam SY Im DS . Lodoxamide attenuates hepatic fibrosis in mice: Involvement of GPR35. Biomol Ther (Seoul). (2020) 28:92–97. doi: 10.4062/biomolther.2018.227, PMID: 31189299 PMC6939691

[146] MacKenzie AE Caltabiano G Kent TC Jenkins L McCallum JE Hudson BD . The antiallergic mast cell stabilizers lodoxamide and bufrolin as the first high and equipotent agonists of human and rat GPR35. Mol Pharmacol. (2014) 85:91–104. doi: 10.1124/mol.113.089482, PMID: 24113750 PMC3868900

[147] Milligan G . Orthologue selectivity and ligand bias: Translating the pharmacology of GPR35. Trends Pharmacol Sci. (2011) 32:317–325. doi: 10.1016/j.tips.2011.02.002, PMID: 21392828

[148] Jenkins L Alvarez-Curto E Campbell K de Munnik S Canals M Schlyer S . Agonist activation of the G protein-coupled receptor GPR35 involves transmembrane domain III and is transduced via Gα_13_ and β-arrestin-2. Br J Pharmacol. (2011) 162:733–748. doi: 10.1111/j.1476-5381.2010.01082.x, PMID: 20958291 PMC3041261

[149] Jenkins L Brea J Smith NJ Hudson BD Reilly G Bryant NJ . Identification of novel species-selective agonists of the G-protein-coupled receptor GPR35 that promote recruitment of β-arrestin-2 and activate gα13. Biochem J. (2010) 432:451–459. doi: 10.1042/bj20101287, PMID: 20919992

[150] GlaxoSmithKline . A first time in human study to evaluate the safety and tolerability of GSK4381406 in healthy participants. Available online at: https://clinicaltrials.gov/study/NCT05999708?term=NCT05999708&rank=1 (Accessed December 15, 2023).

[151] Nestor MS Berman B Fischer DL Han H Gade A Arnold D . A randomized, double-blind, active- and placebo-controlled trial evaluating a novel topical treatment for keloid scars. J Drugs Dermatol. (2021) 20:964–968. doi: 10.36849/jdd.6197, PMID: 34491021

[152] Papp A . FS2 safety and tolerability study in healthy volunteers. Available online at: https://clinicaltrials.gov/study/NCT06807021?term=FS2&rank=1 (Accessed November 17, 2025).

[153] Crossignal Therapeutics I . A study to determine the effect of CT-3001 in patients with advanced solid tumors. Available online at: https://clinicaltrials.gov/study/NCT06598007?intr=GPR35&rank=1 (Accessed November 4, 2024).

[154] Zhao P Sharir H Kapur A Cowan A Geller EB Adler MW . Targeting of the orphan receptor GPR35 by pamoic acid: A potent activator of extracellular signal-regulated kinase and β-arrestin2 with antinociceptive activity. Mol Pharmacol. (2010) 78:560–568. doi: 10.1124/mol.110.066746, PMID: 20826425 PMC2981393

[155] ThirtyFiveBio . Thirtyfivebio stealth emergence. Available online at: https://30fivebio.com/ (Accessed March 30, 2023).

[156] Price ML Wyatt RA Crastin A Jamaluddin A Hardy RS Frost M . G protein-coupled receptor 35 (GPR35) stimulation reduces osteoclast activity in primary human bone cells. JBMR Plus. (2025) 9:ziaf131. doi: 10.1093/jbmrpl/ziaf131, PMID: 40978132 PMC12445851

[157] Gupta RA Higham JP Pearce A Urriola-Muñoz P Barker KH Paine L . GPR35 agonists inhibit trpa1-mediated colonic nociception through suppression of substance p release. Pain. (2025) 166:596–613. doi: 10.1097/j.pain.0000000000003399, PMID: 39382322 PMC11808708

[158] Otkur W Wang J Hou T Liu F Yang R Li Y . Aminosalicylates target GPR35, partly contributing to the prevention of dss-induced colitis. Eur J Pharmacol. (2023) 949:175719. doi: 10.1016/j.ejphar.2023.175719, PMID: 37054942

